# Multi-isotope analysis of mammal bones provides environmental context for the adoption of agriculture in the Tehuacan Valley of Mexico

**DOI:** 10.1126/sciadv.adw9222

**Published:** 2026-03-04

**Authors:** Andrew D. Somerville, Isabel Casar, Rocío Hernández-Flores, Francisco Otero, Edith Cienfuegos Alvarado, Daniel Dalmas, Joaquín Arroyo-Cabrales, Kent V. Flannery, Pedro Morales-Puente, Laura E. Beramendi-Orosco

**Affiliations:** ^1^ Department of World Languages and Cultures, Iowa State University, Ames, IA, USA.; ^2^ Departamento de Estado Solido, Instituto de Física, Universidad Nacional Autónoma de México, Ciudad de México, México.; ^3^ Facultad de Ciencias Políticas y Sociales, Universidad Nacional Autónoma de México, Ciudad de México, México.; ^4^ Laboratorio de Isótopos Estables – LANGEM, Instituto de Geología, Universidad Nacional Autónoma de México, Ciudad de México, México.; ^5^ Department of Anthropology, The University of Utah, Salt Lake City, UT, USA.; ^6^ Subdirección de Laboratorios y Apoyo Académico, Instituto Nacional de Antropología e Historia, Ciudad de México, México.; ^7^ Museum of Anthropological Archaeology, The University of Michigan, Ann Arbor, MI, USA.

## Abstract

The domestication and global spread of maize (*Zea mays*) are pivotal processes in world history. Yet, despite the current importance of maize in global nutrition, food security, and trade, much of its origins remain debated. This paper addresses environmental explanations for the initial intensification of maize agriculture by reconstructing the paleoenvironment of the Tehuacan Valley, Puebla, Mexico. Multi-isotope analysis (δ^13^C_ap_, δ^18^O_ap_, δ^13^C_col_, and δ^15^N_col_) of deer (*Odocoileus virginianus*) and rabbit (*Sylvilagus* spp.) bones from archaeological deposits produces proxy environmental data relevant to the botanical composition of the landscape, precipitation, and temperature. Results indicate that maize cultivation began in the Tehuacan Valley during a relatively wet period, while agricultural intensification and social complexity emerged several centuries later, during a possible dry/wet transition. Stable isotope results are contextualized within broader paleoclimate and archaeological records. This study enhances our understanding of the environmental setting in which agricultural intensification first occurred in Mesoamerica and contributes to discussions on the origins of farming more broadly.

## Teaser

Isotopic values of animal bones reflect environmental conditions during the origins of agriculture in south-central Mexico.

## INTRODUCTION

Maize, or corn [*Zea mays* subspecies (spp.) *mays*], is one of the most important agricultural commodities in the world. Recent advances in genetic and archaeological research have improved our understanding of the ancient history of maize, demonstrating that it was domesticated from a lowland annual grass species, teosinte (*Z. mays* spp. *parviglumis*), sometime before 9000 calibrated years before present (cal BP) in southwestern Mexico (*1*, *2*). After its domestication, maize spread rapidly across the Mesoamerican tropics and beyond (*3*), reaching South America by ~6500 cal BP (*4*) and North America by ~5600 cal BP (*5*). Approximately 3000 years after the earliest evidence for domestication, maize reached the highlands of Mexico, where it hybridized with a highland species of teosinte (*Z. mays* spp. *mexicana*) sometime between 6000 and 4000 cal BP, giving it new adaptive potential to the cooler and drier environments of the highlands (*6*–*8*). Archaeological studies indicate that between approximately 4500 to 3500 cal BP, maize farming became widely practiced across North, Central, and South America, supporting the growth of human populations and enabling the development of complex political and economic systems (*9*–*11*). Despite these advances in maize research, gaps remain in our understanding of the incentives that led hunter-gatherers-cultivators to spread maize so broadly and, eventually, to abandon their former subsistence strategies and embrace farming lifestyles.

Because of the large climatic shifts that characterized the Pleistocene-Holocene transition (~11,700 cal BP), climatic or environmental changes are often proposed as explanations for the initial domestication of plants across the world during the Early Holocene (*12*). Although climatic changes account for the global parallels and may serve as a satisfactory macroscale explanation for the adoption of agriculture, there is little agreement on the exact role that climate played in the process. While some argue that negative environmental changes such as drought or population pressure pushed humans to increasingly rely on cultivated plants, such as maize, in particular regions of the world (*13*–*15*), others posit that climatic amelioration and stability enabled or pulled humans to exploit particular resources more effectively (*16*–*18*). To test environment-based hypotheses for the adoption of agricultural lifeways, reconstructions of the paleoenvironmental context are essential.

**Fig. 1.**
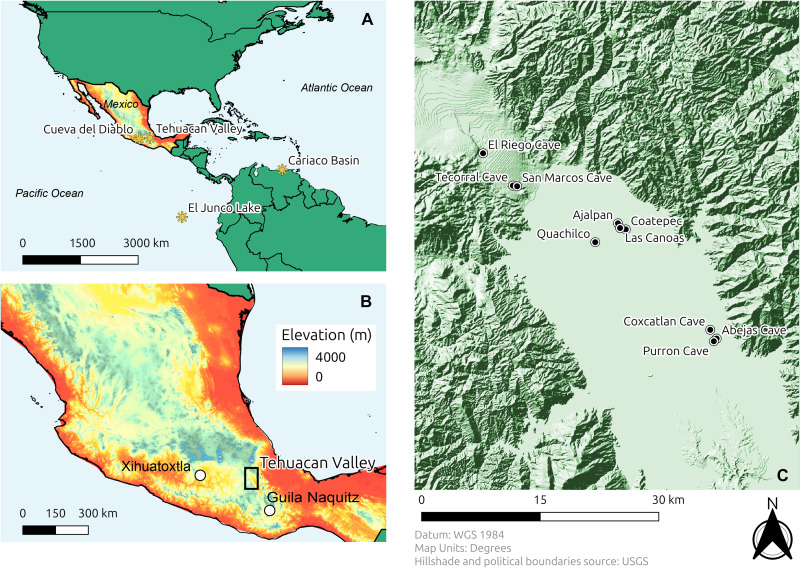
Map of the Tehuacan Valley. (**A**) Location of the Tehuacan Valley and paleoclimate study sites mentioned in the text. (**B**) Location of the Tehuacan Valley, Guila Naquitz Cave, and the Xihuatoxtla rock shelter within Mexico. (**C**) Location of archaeological sites in the Tehuacan Valley from which faunal bones were sampled.

Here, we explore the environmental setting in which people first adopted maize agriculture in the Tehuacan Valley of south-central Mexico in the modern state of Puebla (Fig. 1). Because of its rich biodiversity of plants and animals and because of its long and well-documented history of human occupation, the Tehuacan Valley is today part of The United Nations Educational, Scientific and Cultural Organization World Heritage Site, “Tehuacán-Cuicatlán Valley: Originary Habitat of Mesoamerica.” The semi-arid climate and presence of dry caves in this highland valley (~1000 to 1700 m above sea level) have resulted in the exceptional preservation of organic materials, enabling morphological and genetic studies of their remains (*19*–*22*). Research in the valley has uncovered some of the earliest macrofossil remains of maize in the world, dating to ~5400 cal BP (*23*, *24*) and some of the largest and earliest irrigation structures in the Americas (*25*). In addition to the plant and animal remains, the rich archaeological record of the Tehuacan Valley documents the presence of early hunter-gatherers, seminomadic mixed-strategy horticulturalists, and sedentary agricultural societies with complex political organizations (*26*–*28*). The exceptional preservation and long temporal sequence make the Tehuacan Valley an opportune location to study the long-term dynamics of human-environmental interactions and to investigate the processes of agricultural adoption and intensification.

To provide information about the paleoenvironmental context, we conduct a multi-isotope analysis (δ^13^C_ap_, δ^18^O_ap_, δ^13^C_col_, and δ^15^N_col_) of bone (bioapatite and collagen) from deer (*Odocoileus virginianus*) and rabbit (*Sylvilagus* spp.) specimens recovered from archaeological investigations in the Tehuacan Valley. Isotope values of herbivore skeletal tissues reflect the isotopic ratios of the plants and water consumed by the organisms while alive (*29*–*31*). Assessments of isotopic ratios of herbivore bones can therefore provide information about the environments in which the organisms lived, including the botanical composition of the landscape, precipitation, humidity, and temperature (*32*–*37*). For more background on the principles of stable isotope analysis and paleoenvironmental reconstruction, please see Supplementary Text.

The sample is comprised of faunal bones recovered from stratigraphically excavated contexts with associated artifacts and directly dated maize specimens. Age ranges of the cultural phases are estimated through Bayesian modeling of available radiocarbon dates (Table 1 and table S1) (*20*, *23*, *38*–*40*). Stable isotope data from each bone specimen therefore can be associated with the contemporaneous record of material culture and botanical remains and can be situated within regional and global paleoclimatic records. This research allows us to reconstruct characteristics of the paleoenvironment during the transition to maize farming and is relevant to ongoing debates on the adoption of agriculture in Mesoamerica and across the globe more broadly.

**Table 1.** Revised chronological sequence of the Tehuacan Valley. Phase, period, and subepoch divisions are synthesized from results of previous studies (*20*, *38*, *40*, *42*) and informed by results of new Bayesian modeling of radiocarbon dates (Supplementary Text and data S1). Human subsistence strategies are inferred from the archaeological record (*39*).

Approx. dates (cal BP)	Approx. dates (BC/AD)	Cultural phases	Regional periods	Geological subepoch	Subsistence strategies
1250–450	AD 700–1521	Venta Salada	Epiclassic/Postclassic	Late Holocene	Sedentary agriculture
2250–1250	300 BC–AD 700	Palo Blanco	Classic		
2800–2250	850–300 BC	Santa Maria	Formative		
3250–2800	1300–850 BC	Ajalpan			
4550–3250	2600–1300 BC	Purron			Mixed horticulture-foraging-hunting strategy, semisedentary
6300–4550	4350–2600 BC	Abejas	Archaic	Middle Holocene	
7900–6300	5950–4350 BC	Coxcatlan			
9900–7900	7950–5950 BC	El Riego		Early Holocene	Hunting and gathering, seasonal mobility
33,000–9900	31,000–7950 BC	Ajuereado	Paleoindian	Late Pleistocene	Human occupation uncertain

## RESULTS

### Chronology

Chronological modeling of 94 available radiocarbon ages, including 15 directly dated animal bone samples, allowed the isotopic data from each bone specimen of the present study to be assigned to one of nine pre-Colonial cultural phases (data S1, fig. S1, and Table 1). Notably, our Bayesian radiocarbon model differs in several ways with the traditional chronological sequence for the valley presented by Johnson and MacNeish (*38*) (Supplemental Text). We use this revised chronology to situate the isotopic data within regional and global paleoclimate records.

### Species differences

Deer and rabbit bone specimens were analyzed from 10 archaeological sites within the Tehuacan Valley (table S2). Initial diagenesis tests of bone samples found that 176 bone specimens (99 rabbits and 77 deer) yielded viable apatite data and 81 specimens (59 rabbits and 22 deer) yielded viable collagen for stable isotope analysis. Stable isotope and diagenesis testing results are presented in data S2. Although there was an overlap in stable isotope values of deer and rabbit specimens, some significant differences were found between the taxa. Welch’s *t* tests documented significant differences between the deer and rabbits means of δ^13^C_ap_ values (*t* = −3.753, df = 173.95, and *P* < 0.001) and δ^18^O_ap_ values (*t* = −4.4686, df = 131, and *P* = 0.001), but not of δ^13^C_col_ values (*t* = −1.8933, df = 52.373, and *P* = 0.06385) or δ^15^N_col_ values (*t* = 1.4409, df = 33.694, and *P* = 0.1589). In general, rabbits tended to have higher δ^13^C_ap_ and δ^18^O_ap_ values and lower δ^15^N_col_ values than deer, indicating that rabbits consumed more C4 or crassulacean acid metabolism (CAM) plants and inhabited drier patches of the landscape than did deer. The consistent differences between rabbits and deer reflect the different ecological niches that these two taxa filled within the valley. The lower δ^15^N_col_ values from rabbits may also be influenced by their digestive strategy of coprophagy, passing fibrous food twice through the digestive tract, potentially resulting in lower diet-tissue offsets in δ^15^N_col_ values (*37*). The descriptive statistics of the results by genera are listed in table S3.

Within the rabbit sample, two morphotypes were observed: large cottontails and small cottontails. Discussions of rabbit classification and isotopic analyses are presented in greater detail in the Supplementary Text. Large cottontails (cf. *Sylvilagus cunicularius*) had significantly higher δ^13^C_ap_ (*t* = 5.7159, df = 72.057, and *P* < 0.001) and δ^13^C_col_ values (*t* = 4.7888, df = 35.107, and *P* < 0.001) than small cottontails (*Sylvilagus audubonii* or *Sylvilagus floridanus connectens*), likely reflecting the greater tolerance of open grasslands by the larger *S. cunicularius* than the smaller cottontails. No significant differences were observed between cottontail species in terms of δ^18^O_ap_ values (*t* = −0.61742, df = 73.493, and *P* = 0.5389) or δ^15^N values (*t* = 1.6733, df = 38.688, and *P* = 0.1023). While *S. cunicularius* specimens exhibit higher δ^13^C_ap_ and δ^13^C_col_ values trend toward higher δ^15^N_col_ values, this pattern becomes inverted during the Santa Maria and Venta Salada phases, in which *S. cunicularius* specimens exhibited lower values than the smaller cottontails. This suggests different environmental conditions or different methods of hunting or meat acquisition by humans during these phases. However, because of the overall similar diets and habitats of different rabbit species, their overlapping habitat ranges (*41*), and because previous studies have suggested that rabbit species register environmental-influenced stable isotope ratios in the skeletal tissues according to similar principles (*37*), all rabbits are combined for the paleoenvironmental analysis of this paper. The “rabbits” category, then, represents the general rabbit niche, which includes at least two cottontail species with slightly different habitat preferences. Nonetheless, as an added level of granularity in the analysis we present descriptive statistics of isotopic results from different rabbit morphotypes in table S4 and visualize their changes over time in fig. S2.

### Inter epoch comparisons

**Fig. 2.**
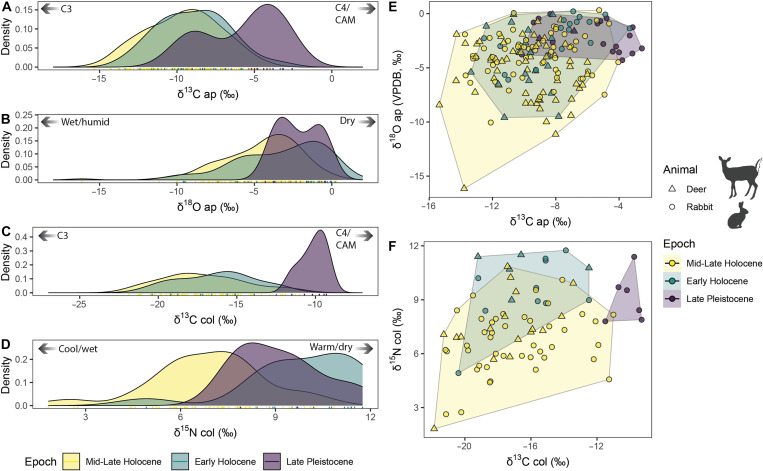
Density and scatterplots of stable isotope values from animal bone apatite and collagen samples. (**A**) Density plot of stable oxygen isotope values in deer and rabbit bone bioapatite. (**B**) Density plot of stable carbon isotope values in deer and rabbit bone bioapatite. (**C**) Density plot of stable carbon isotope values in deer and rabbit bone collagen. (**D**) Density plot of stable nitrogen isotope values in deer and rabbit bone collagen. (**E**) Scatterplot of stable carbon and oxygen isotope values in bone bioapatite. (**F**) Scatterplot of stable carbon and nitrogen isotope values in bone collagen. Marker shapes differ by animal genus. Silhouette images accessed through PhyloPic: *Sylvilagus audubonii* by G. Montgomery, licensed under CC BY 4.0 (https://creativecommons.org/licenses/by/4.0/deed.en); *Odocoileus virginianus* by G. Palomo-Muñoz, licensed under CC BY-NC 3.0 (https://creativecommons.org/licenses/by-nc/3.0/deed.en).

Herbivore bone isotope values permit comparisons of environmental conditions during the Late Pleistocene and Holocene epochs. We investigated the stable isotope results according to the subepochs (Late Pleistocene, Early Holocene, Middle Holocene, and Late Holocene), following the formal Holocene subdivisions of Walker *et al.* (*42*), and by the cultural phases within the valley (Table 1). For the subepoch comparisons, we used an analysis of variance (ANOVA) to test for differences between isotope variable means, finding significant differences for δ^13^C_ap_ [*F*(3, 171) = 16.4, *P* < 0.001], δ^18^O_ap_ [*F*(3, 171) = 6.077, *P* < 0.01], δ^13^C_col_ [*F*(3, 77) = 13.06, *P* < 0.001], and δ^15^N_col_ [*F*(3, 77) = 11.48, *P* < 0.001] values. Post hoc Tukey’s tests of honest significant difference (HSD) found that the most significant differences occurred between the Late Pleistocene and the other subepochs (table S5). Because bone isotopic values from Middle and Late Holocene contexts were similar and overlapping with the only significant differences being between mean δ^15^N_col_ values (*P* = 0.044) (table S5) and because the periods are sequential in time, we combined these two subepochs them into a single category for visualization (Fig. 2, A to D).

### Cultural phases of the Tehuacan Valley

**Fig. 3.**
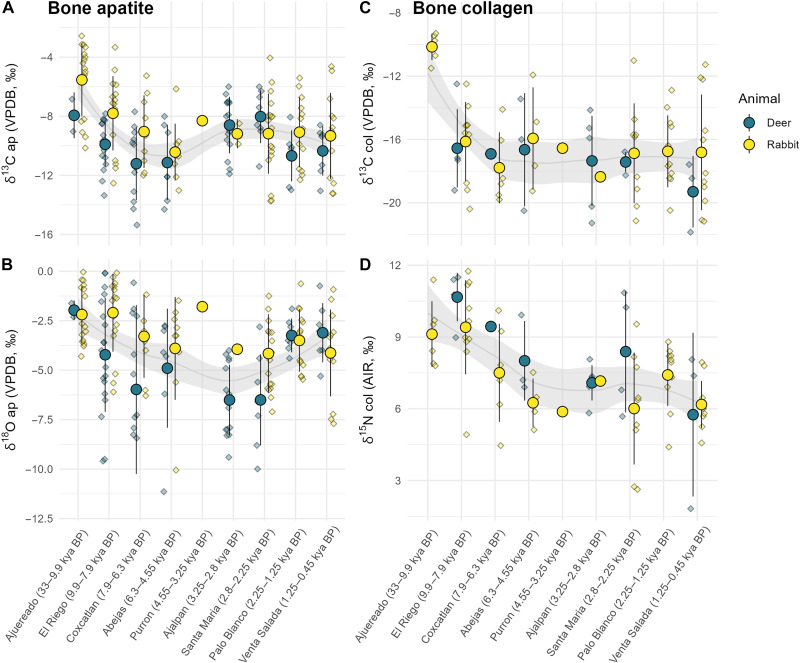
Strip plots of stable isotope values from animal bone apatite and collagen. Plots are grouped by animal type and cultural phase. Large circles represent phase means and small diamonds represent individual bone samples. Error bars denote 1 SD. The shaded gray area around the regression line indicates the 95% confidence interval band generated with a locally estimated scatterplot smoothing (LOESS) function using the pooled deer and rabbit sample. All dates are presented according to calibrated radiocarbon ages. (**A**) Stable carbon isotope values from bone apatite carbonate. (**B**) Stable oxygen isotope values from bone apatite carbonate. (**C**) Stable carbon isotope values from bone collagen. (**D**) Stable nitrogen isotope values from bone collagen.

One-way Welch’s ANOVA tests were used to explore differences between the isotope means of the cultural phases for all faunal specimens. The Purron phase (~4550 to 3250 cal BP) was excluded from statistical tests because it was represented by only a single rabbit bone. Significant differences were found in stable isotope values between the phases for δ^13^C_ap_ [*F*(7, 66.272) = 6.825, *P* < 0.001], δ^18^O_ap_ [*F*(7, 66.598) = 8.995, *P* < 0.01], δ^13^C_col_ [*F*(7, 26.98) = 28, *P* < 0.001], and δ^15^N_col_ values [*F*(7, 26.382) = 6.153, *P* < 0.001]. When focusing only on rabbit bone isotope values, significant differences were also found between phases (*P* < 0.01 for all). When focusing on deer, significant differences were found between phases for δ^18^O_ap_ values (*P* < 0.001) and δ^15^N_col_ values (*P* = 0.014), but not for δ^13^C_ap_ values (*P* = 0.056) or δ^13^C_col_ values (*P* = 0.720). The differences between cultural phases are visualized in the grouped strip plots of Fig. 3 (A to D). Differences between the phases are assessed qualitatively and descriptively according to each isotope variable below.

### Carbon isotope values over time

The δ^13^C_ap_ values of both deer and rabbits show a steady decrease across the Late Pleistocene, the Early Holocene, and Middle Holocene contexts (i.e., Ajuereado, El Riego, Coxcatlan, and Abejas phases) (Fig. 3A). The specimens from the oldest archaeological deposits within the valley (Ajuereado phases) exhibit the highest mean values δ^13^C_ap_ values for both rabbits (*x̄* = −5.5 ± 2.5‰) and deer (*x̄* = −7.9 ± 1.5‰). During the Abejas phase, both rabbits (*x̄* = −10.4 ± 1.9‰) and deer (*x̄* = −11.2 ± 2.5‰) exhibit relatively low δ^13^C_ap_ values, suggesting that the landscape had seen an increase in C3 plants relative to C4/CAM plants during the first several millennia after the Pleistocene-Holocene transition. After the Coxcatlan and Abejas phases of the Middle Holocene, the leporid δ^13^C_ap_ values do not exhibit a clear pattern within the Late Holocene, but the deer specimens from the Ajalpan and Santa Maria phases exhibit significantly higher δ^13^C_ap_ values than the mean δ^13^C_ap_ values from deer bones in the subsequent Palo Blanco and Venta Salada phases. The single rabbit bone from the Purron phase exhibits a relatively high δ^13^C_ap_ value of −8.3‰. In general, the mean values of stable carbon isotope variables from rabbit bones remain fairly consistent across the Late Holocene, changing from −9.3 ± 2.3‰ in the Palo Blanco phase to −9.2 ± 2.7‰ in the Santa Maria phase, to −9.3 ± 2.9‰ in the Venta Salada phase.

Stable carbon isotope values from bone collagen exhibit less temporal patterning than the values from bone apatite carbonate (Fig. 3C). Nonetheless, the highest mean δ^13^C_col_ values are from rabbit specimens associated with Ajuereado phase deposits (*x̄* = −10.1 ± 0.8‰), similar to the trend observed with the δ^13^C_ap_ data and supporting the interpretation that the Late Pleistocene environment included a greater abundance of C4 or CAM plants relative to conditions during the Holocene. No deer collagen samples were available from Ajuereado, Purron, or Palo Blanco phase deposits.

### Oxygen isotope values over time

Rabbits from the El Riego phase display the highest average δ^18^O_ap_ value (*x̄* = −2.1 ± 2.0‰) of the sequence, slightly higher than rabbits from the preceding Ajuereado phase (*x̄* = −2.2 ± 1.4‰). These values suggest that both the Ajuereado and El Riego phases were characterized by low precipitation and low humidity, and that the El Riego phase was likely the driest phase of the sequence. Oxygen isotope values from the subsequent Coxcatlan and Abejas phases are significantly depleted in ^18^O in both leporid and deer tissues, suggesting that precipitation and humidity increased during this period, which corresponds to the Middle Holocene. The singular rabbit bone from the Purron phase exhibits a relatively high δ^18^O_ap_ value of −1.8‰, suggesting low precipitation. Deer δ^18^O_ap_ values from the following two phases, Ajalpan and Santa Maria, exhibit the lowest δ^18^O_ap_ means of the sample. The lowest mean δ^18^O_ap_ values for both deer (*x̄* = −6.5 ± 2.3‰) and rabbits (*x̄* = −4.2 ± 2.0‰) are from specimens from Santa Maria phase deposits, suggesting that this was the phase with the highest precipitation and humidity of the sample. The last two phases of the sequence (Palo Blanco and Venta Salada) are characterized by relatively high δ^18^O_ap_ values from deer bones, suggesting that these two Classic period phases were characterized as being drier than the preceding two Formative period phases. Rabbit bones display δ^18^O_ap_ values in the Palo Blanco phase (*x̄* = −3.5 ± 1.6‰) that are higher than the preceding Ajalpan and Santa Maria phases, but rabbit bones exhibit a decrease in mean δ^18^O_ap_ values from the Palo Blanco to the Venta Salada (−4.1 ± 2.2‰).

### Nitrogen isotope values over time

A general trend of decreasing δ^15^N_col_ values over time was observed for both deer and rabbit bones. While no deer specimens from Late Pleistocene contexts were available for collagen isotope analysis, the rabbits in the Late Pleistocene (Ajuereado) phase exhibit δ^15^N_col_ values (*x̄* = +9.1 ± 1.4‰) that are higher than every other phase except for the following El Riego phase of the Early Holocene, in which rabbits (*x̄* = +9.4 ± 2.0‰) and deer (*x̄* = +10.7 ± 1.0‰) exhibit the highest means of the sample. This suggests that the Late Pleistocene and the Early Holocene were the warmest or driest phases of the sample. Following the El Riego phase, mean δ^15^N_col_ values of both deer and rabbits steadily decrease across the Coxcatlan and Abejas phases (Fig. 3D). This trend was likely driven by the local environment of the valley becoming more humid and greener across the Middle Holocene. The singular rabbit bone from the Purron phase exhibits a relatively low δ^15^N_col_ value of +5.9‰. The phase with the lowest δ^15^N_col_ values of the sample is the Venta Salada phase, in which deer (*x̄* = +5.8 ± 3.4‰) and rabbit (*x̄* = +6.2 ± 1.0‰) specimens exhibit exceptionally low mean values. This suggests that cooler and/or wetter conditions prevailed at that time. Notably, Venta Salada is the last phase of the valley before Spanish conquest, and the period in which the valley contained the largest human population.

### Mixing model of environmental change over time

**Fig. 4.**
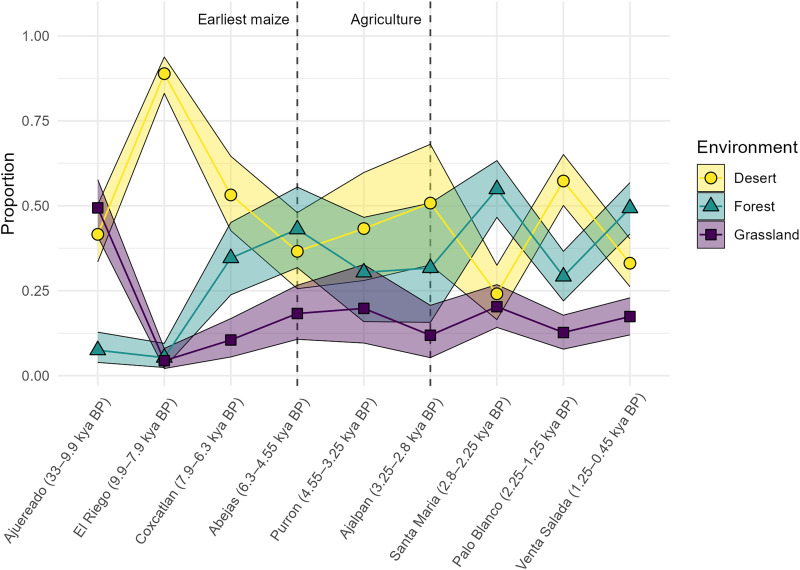
Line plot of the results of the MixSIAR Bayesian mixing model using rabbit bone stable isotope values. The model estimates the proportional contribution of different environmental patches to the diet of rabbits over time, incorporating δ^13^C_ap_, δ^18^O_ap_, δ^13^C_col_, and δ^15^N_col_ values. Markers indicate the estimated median proportion of patch contribution to the diet. Shaded ribbons indicate the interquartile range (25th to 75th percentiles) of the posterior credible distribution. The vertical dashed lines indicate the Abejas phase, the phase with the earliest directly dated maize specimens from the Tehuacan Valley (~5400 cal BP) (*23*) and the Ajalpan phase, the first cultural period with strong evidence of sedentary agriculture.

The results of a Bayesian stable isotope mixing model using isotope values from rabbit bones and generalized environmental parameters (table S6) illustrate paleoenvironmental changes over time (Fig. 4). Rabbits were preferred over deer for the model due the greater availability of isotopic datasets from rabbit bones in comparable ecosystems (*37*, *43*) and due to the greater abundance of rabbits within the Tehuacan collection. The model posteriors, presented fully in table S7, indicate that rabbits from the Late Pleistocene (Ajuereado phase) were feeding within an environment characterized by high proportions of grassland and desert patches (Fig. 4). During the El Riego phase, the first phase of the Holocene, the grassland and forest patches dropped to a near zero proportional contribution to rabbit diets and the desert patch rose to a very high proportion. During the Middle Holocene phases of Coxcatlan and Abejas, the desert proportion dropped over time, and the forest and grassland patches rose, suggesting a change in environmental conditions likely resulting from greater moisture availability during the Coxcatlan and Abejas phases. Although the Purron phase was only represented by a single rabbit mandible, the measured isotopic values hint that the environment may have become drier at that time. The subsequent Ajalpan phase, which also has a small sample size, appears to have been dry, with a high proportion of desert patch. However, the deer samples from the Ajalpan phase, which were not included in this model, exhibit very low δ^18^O values, suggesting environmental complexity at this time. The values from the Santa Maria phase suggest that the Tehuacan Valley had the greatest proportion of C3/forest vegetation during this phase and had the lowest proportion of open desert. The final two phases, Palo Blanco and Venta Salada, alternate between desert and forest as the dominant patches in the diet, suggesting that the Palo Blanco phase was drier than the Epiclassic/Postclassic Venta Salada phase.

### Broader climatic context

**Fig. 5.**
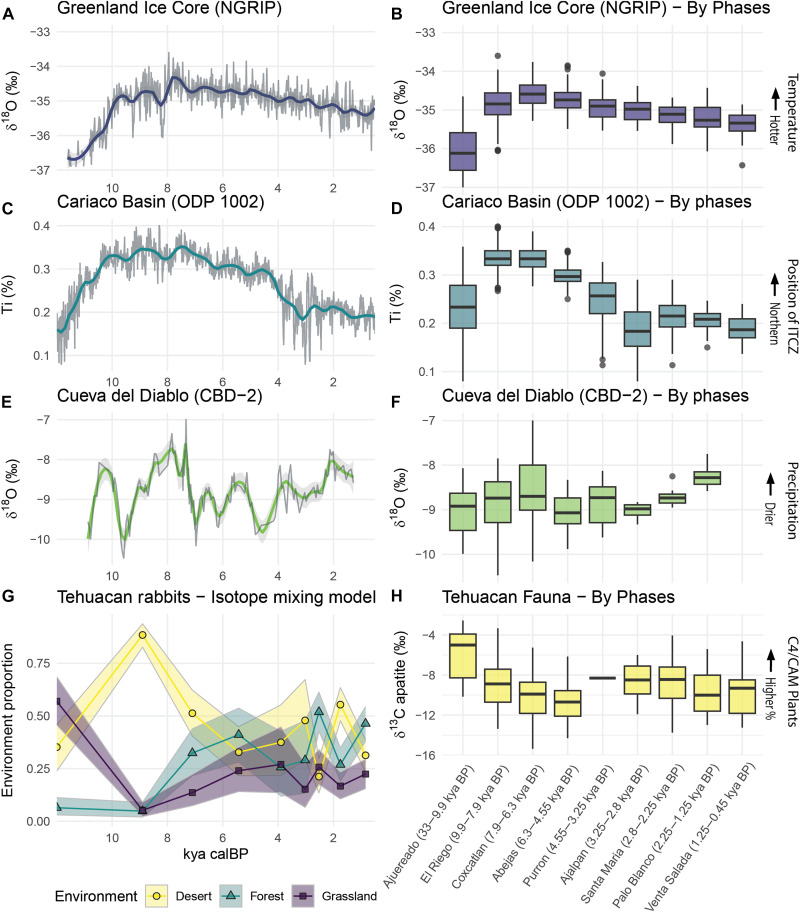
Tehuacan Valley paleoenvironment data plotted with relevant paleoclimate records. Graphs in the left column display continuous paleoclimate proxy plotted against the estimated radiocarbon age (12,000 to 0 cal BP), while graphs in the right column display the data binned according to the cultural phases of the Tehuacan Valley (kya cal BP). (**A** and **B**) δ^18^O values from the Northern Greenland Ice Core (NGRIP) (*45*). Higher values indicate higher temperatures. (**C** and **D**) Ti % from ocean sediment core (ODP 1002) from the Cariaco Basin, Venezuela (*52*). Higher values indicate more northern position of the ITCZ. (**E** and **F**) δ^18^O values from Cueva del Diablo speleothem (CBD-2), Mexico (*50*). The values correlate with summer precipitation levels with higher values indicating drier summers and lower values indicating wetter summers. (**G**) Stable isotope mixing model results from analysis of values from Tehuacan rabbit bones. (**H**) Stable carbon isotope values from Tehuacan Valley deer and rabbit bone specimens.

The stable isotope results are better understood when contextualized within the broader paleoclimatic record. Because human behavior responds to variables at multiple scales, we present the broader climatic data as continuous and binned variables according to the cultural phases of the Tehuacan Valley (Fig. 5).

Globally, stable oxygen isotope values from the Greenland ice cores (Fig. 5, A and B) indicate that the Pleistocene climate was characterized as cooler, drier, and more variable than the Holocene (*44*, *45*), a trend supported by diatom evidence from lake sediment cores from central Mexico (*46*, *47*). The faunal isotope values from Tehuacan Valley indicate high amounts of C4/CAM vegetation in the diet of herbivores and suggest that it was a generally dry period characterized by extensive grasslands.

After the transition from the Pleistocene to the Holocene, the climate globally became warmer and more stable in temperature. However, within the first several millennia after the Younger Dryas, the Early Holocene experienced a global climatic disturbance characterized by widespread aridity that may have been due to the influx of cold, freshwater into the Atlantic from melting glaciers at approximately 8200 cal BP (*48*, *49*). In Mexico, this dry event is evidenced by lower lake levels and greater lake salinity in studied lakes of central Mexico (*47*) and reduced monsoon precipitation in southwestern Mexico (*50*). The 8200 cal BP dry event happened during the El Riego phase in the Tehuacan Valley (~9900 to 7900 cal BP), which the faunal isotope data indicate was the driest period of the sequence.

The primary source of precipitation for the Tehuacan Valley is the summer monsoon brought by the seasonal northern migration of the intertropical convergence zone (ITCZ), a low-pressure belt encircling the earth near the equator that causes rising air and precipitation (*51*). Following the aridity of the Early Holocene, the Holocene Thermal Maximum (HTM) occurred during the Middle Holocene, a time that appears to have been wetter and warmer in Mexico, with a more northern position of the ITCZ (Fig. 5, C and D) and hence more precipitation (*52*, *53*). Paleoclimate proxy data, particularly ostracod δ^18^O values from lake sediments in southern Mexico (*54*), suggest that the peak of the HTM coincided with the ITCZ reaching its northernmost position during the Holocene at around 7300 to 7100 cal BP, a period that was characterized by more humid conditions and greater monsoon activity in southern and central Mexico (*50*, *54*). The faunal isotope data from the Tehuacan Valley during the Coxcatlan (7900 to 6300 cal BP) and Abejas (6300 to 4550 cal BP) phases indicate that these phases were wetter and contained more C3 vegetation than during the preceding Late Pleistocene and Early Holocene (Figs. 3 and 4). These data are in congruence with a more northern position of the ITCZ and greater precipitation during the Middle Holocene.

After the HTM, titanium concentration (Ti %) data from the Cariaco Basin marine sediment core demonstrate a general southward migration of the ITCZ over the course of Late Holocene (*52*) (Fig. 5, C and D). At approximately 4200 cal BP, the Cariaco Basin data record an abrupt southern migration of the ITCZ and a period of increased variability of the El Niño Southern Oscillation (ENSO) and a stronger El Niño in general [(*52*), page 1306]. Sand grain size analysis of a lake core from El Junco Crater Lake in the Galapagos Islands similarly documents an increase in the magnitude or frequency of the ENSO after 4200 cal BP (*55*). In central Mexico, the presence of El Niño conditions typically results in reduced precipitation as ENSO activity can shift the ITCZ southward and cause enhanced subsidence in northern Mexico (*56*), although variation in sea surface temperatures likely mediates the impact (*57*). More broadly, this period corresponds to a known global climatic disturbance from 4200 to 3800 cal BP, which was characterized by widespread aridity in the northern hemisphere (*47*, *49*, *58*–*60*). Unfortunately, only a single rabbit bone from the Tehuacan Valley was available for analysis from this period, the Purron phase (~4550 to 3250 cal BP).

In the Late Holocene, multiple paleoclimate records demonstrate generally wetter conditions during the Late Formative period (*50*, *61*, *62*), which correspond to the Santa Maria phase in our sample. Analyses of δ^18^O values of carbonate from a sediment core from lake Aljojuca Cuenca Oriental of Mexico indicate a particularly wet period from approximately 3000 to 1450 cal BP [(*63*), page 1694]. After approximately 3500 cal BP, speleothem data from the Cueva del Diablo speleothem in southwestern Mexico suggest a few centuries of increased monsoon precipitation until approximate 2200 cal BP (*50*) (Fig. 5, E and F). Within the Tehuacan valley, faunal isotope results from the Santa Maria phase (2800 to 2250 cal BP) indicate that it was an exceptionally wet period, while the subsequent Classic Palo Blanco phase (2250 to 1250 cal BP) was more arid, and the Epiclassic/Postclassic Venta Salada phase (1250 to 429 cal BP) returned to wetter conditions (see Fig. 5, G and H). At the end of the Mesoamerican Classic period, or the Epiclassic period, central and southern Mexico experienced a well-documented megadrought from roughly ~1300 to 1050 cal BP (*54*, *62*–*65*). This drought divides the Palo Blanco and Venta Salada phases in our sample.

## DISCUSSION

### Paleoenvironment of the Tehuacan Valley

At the broadest temporal scale, the isotopic results of this study reflect differences between the paleoenvironment of the Tehuacan Valley in the Late Pleistocene and the Holocene epochs. High carbon, oxygen, and nitrogen stable isotope values in deer and rabbit bones from Ajuereado phase contexts indicate that the Late Pleistocene was dry and characterized by open, C4 grasslands. This interpretation is supported by the faunal record, which shows strong differences in the mammal species between the epochs. In Ajuereado phase contexts, Flannery identified the bones of Pleistocene horses and pronghorn, which are not found in any Holocene contexts (*66*), and significantly different proportions of rodent species between Late Pleistocene and Holocene layers (*66*, *67*). Together the isotopic and faunal data strongly support the notion that the Tehuacan Valley during the Late Pleistocene was drier than today and characterized by widespread grasslands. This grassland environment would have been the setting in which the first humans arrived in the valley, although the exact timing of arrival remains debated (*40*).

**Fig. 6.**
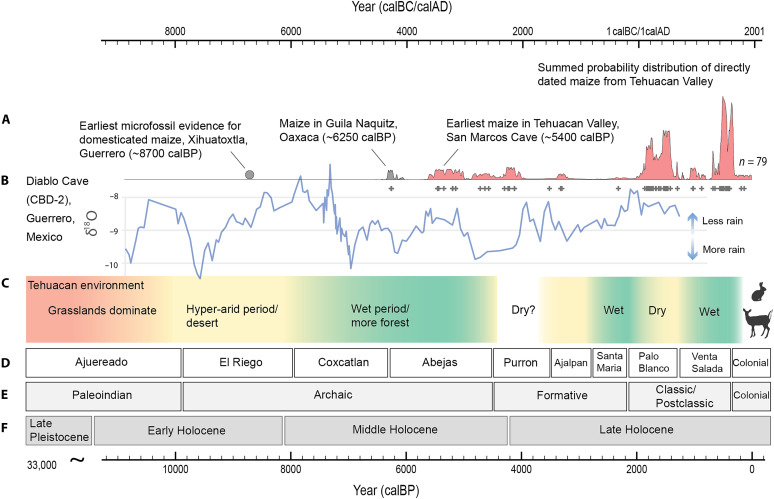
Paleoenvironmental and paleoclimatic data plotted in relation to the timing of maize cultivation in the Tehuacan Valley. (**A**) Summed probability distributions of 79 previously published AMS radiocarbon ages of maize from the Tehuacan Valley (*19*, *23*, *24*) plotted with the earliest macrofossil evidence of maize in the Mexican highlands (*68*) and the date of earliest microfossil evidence for domesticated maize in Mexico (*2*). (**B**) Stable oxygen isotope data as proxy for precipitation from a speleothem from Diablo Cave, Mexico (*50*). (**C**) Interpretation of paleoenvironment in the Tehuacan Valley using the faunal isotopic data of the present study. (**D**) Cultural phase chronology based on previous studies (*20*, *38*, *40*, *95*) and on Bayesian modeling of the present study (data S1, Supplementary Materials, and Table 1). (**E**) Regional cultural phases of highland Mesoamerica. (**F**) Geologic subepochs with Holocene divisions following Walker *et al.* (*42*). Silhouette images accessed through PhyloPic: *Sylvilagus audubonii* by G. Montgomery, licensed under CC BY 4.0 (https://creativecommons.org/licenses/by/4.0/deed.en); *Odocoileus virginianus* by G. Palomo-Muñoz, licensed under CC BY-NC 3.0 (https://creativecommons.org/licenses/by-nc/3.0/deed.en). Images modified for clarity.

Across the first several phases of the Holocene, the animal isotope data demonstrate a clear pattern of change. After a peak in aridity during the Early Holocene El Riego phase (9900 to 7900 cal BP), the steadily decreasing carbon, oxygen, and nitrogen stable isotope values suggest that the environment during the Middle Holocene became wetter and transitioned to a higher ratio of C3 plants, such as mesquite trees and shrubs, relative to C4/CAM plants, such as grass and cacti. This trend, coinciding with the HTM, continued through the Coxcatlan (~7900 to 6300 cal BP) and Abejas (~6300 to 4550 cal BP) phases. The environment may have been more variable during the Late Holocene, as the isotopic data suggest that there were shifts between drier phases (Purron, Ajalpan, and Palo Blanco) and wetter phases (Santa Maria and Venta Salada) (Figs. 4 and 6).

### Paleoenvironment and arrival of maize in the highlands

This multi-isotope analysis of mammal bones from the Tehuacan Valley allows us to examine long-term trends of environmental and social change over the periods in which maize first arrived in the valley and when agriculture became the dominant subsistence strategy. After the domestication of maize from teosinte *parviglumis* in the seasonal lowlands of western Mexico sometime before ~9000 cal BP (*2*), maize cultivation spread rapidly (*3*), reaching South America as early as ~6500 cal BP (*4*) and North America by ~5600 cal BP (*5*). Within Mexico, maize first reached the semi-arid highlands sometime around 6250 cal BP, as evidenced by directly dated maize cobs from the highland site of Guila Naquitz Cave in Oaxaca (*68*, *69*), and was adopted by human populations in the Tehuacan Valley around 5400 cal BP.

Modern domesticated maize differs morphologically from teosinte in several key respects. Maize has a nondisarticulating rachis, while teosinte has a brittle rachis that disarticulates upon maturity; maize has exposed (naked) kernels while teosinte’s kernels are encased in a hard outer glume; maize has paired mature spikelets instead of only a single spikelet; maize ears are polystichous (greater than two ranks), while teosinte is distichous (two ranks); maize is less bushy with reduced axillary branching in comparison to teosinte (*70*); and maize exhibits different root morphology than teosinte, including increased seminal root number (*71*, *72*). The earliest three maize specimens excavated from Guila Naquitz Cave display morphological features associated with modern domesticated varieties, including a nondisarticulating rachis and exposed kernels, but retain teosinte-like features, including small, distichous (two-ranked) cobs with two (*n* = 2) or four (*n* = 1) rows (*73*). Only the singular four-rowed specimen from the early Guila Naquitz cobs exhibits paired spikelets, which is a trait of domesticated maize. The extent of axial branching and root structure of the earliest Guila Naquitz specimens remains unknown.

Although approximately 700 to 800 years later than the first evidence for domesticated maize at Guila Naquitz, the earliest maize specimens from the Tehuacan Valley shared many morphological similarities with the Guila Naquitz maize, including small cob size, nondisarticulating rachis, and exposed kernels (*73*). Most maize cob specimens from the lowest levels of San Marcos Cave, the cave with the oldest evidence for maize in the valley, exhibit eight rows of kernels; however, a single cob had four rows with paired spikelets, demonstrating that it was already domesticated but retained a primitive form [(*74*), page 179; (*75*)]. Recent research on the root morphology of maize specimens from San Marcos Cave dating to ~5100 cal BP shows the presence of multiseriate cortical sclerenchyma and reduced nodal root number, which are traits associated with modern maize, but at least one individual (SM11) completely lacked seminal roots, which is a teosinte-like trait (*71*, *72*). Together, the morphological data on maize from the Tehuacan Valley indicate that the earliest maize was already domesticated as it would have required human assistance in the reproductive cycle (*75*). The absence of a fully expressed suite of domestication traits, however, suggests that early Tehuacan maize represents an early stage in a protracted process of domestication and subsequent improvement rather than a fully stabilized domesticated form.

Genomic analyses of early Tehuacan maize specimens similarly indicate a mosaic process of evolution (*22*, *76*). Studies of the earliest maize specimens in the valley find that some alleles characteristic of modern maize (*td1* = thick tassel dwarf1, *tb1* = teosinte branched1, and *ba1* = barren stalk1) were already fixed in the population, while others alleles (*zagl1* = zea agamous-like1, *su1* = sugary1, and *wx1* = waxy starch1) retained teosinte-like forms (*22*, *76*). Additional genomic research indicates that early domesticated maize from San Marcos Cave in the Tehuacan Valley had already experienced gene flow from highland teosinte (*Zea mays ssp. mexicana*) (*6*), likely facilitating adaptation to the cooler, drier, and more light-intense environments of the Mexican highlands (*7*). Indeed, Yang *et al.* (*6*) found admixture from highland teosinte *mexicana* in every domesticated maize genome they analyzed (*n* > 1000), with the exception of a ~5500 cal BP specimen from northern Peru (*77*). These findings highlight the importance of the Mexican highlands in the process of maize domestication, and underscore the significance of the Tehuacan Valley, located within the natural range of teosinte *mexicana*, as a key region for understanding the secondary improvement of domesticated maize.

According to the results of our study, the oldest directly dated specimens of domesticated maize in the Tehuacan Valley appear during the Abejas phase, a particularly wet interval of the Middle Holocene (Fig. 6, A to C). The earliest cobs came from excavations of San Marcos Cave and include specimens AA-No. 3305 (4700 ± 60 BP) and AA-No.3311 (4700 ± 110 BP), which calibrate to the mean (±SD) ages of 5432 ± 80 cal BP and 5419 ± 144 cal BP, respectively (*23*). An additional cob without contextual information (Tehuacan162) dates to the Abajas phase with a mean radiocarbon age of 5132 ± 100 cal BP (Beta-365324 = 4460 ± 30 BP) (*76*). More recent excavations and research at San Marcos Cave discovered and directly dated the oldest maize specimens to a similar chronological age of about 5100 cal BP (Beta-320310 = 4480 ± 30, Beta-320309 = 4450 ± 30) (*22*, *24*). These results indicate that humans first brought domesticated maize to the valley during the middle of the Abejas phase (6300 to 4550 cal BP) (*23*, *24*), a time that the faunal isotope data suggest was exceptionally wet and high in C3 vegetation (Fig. 6). The earlier highland maize specimens from Guila Naquitz similarly date to the Abejas phase, and together, data from these sites suggest that humans first brought maize to the highlands during a period in which wetter environmental conditions prevailed. This relatively favorable climatic window may help explain why early maize, without the full suite of domestication traits, could succeed under initial cultivation in the highlands.

### Paleoenvironment and agricultural intensification

Despite its adoption by human populations in the highlands during the Abejas phase, maize cultivation remained at a low-level of production for several more centuries (*78*), likely forming part of a seasonal subsistence regime. Across Mesoamerica, the Formative period (~4500 to 2200 cal BP) marked the widespread adoption of maize agriculture, reductions in seasonal mobility, and the emergence of village-based farming communities (*10*, *26*). In the Tehuacan Valley, the initial phase of the Formative period, the Purron phase (~4550 to 3250 cal BP), coincided with a known period of aridity documented across much of the Northern Hemisphere between ~4200 and 3800 cal BP (*49*). So widespread and recognized was this drought that its presence formerly divides the Middle and Late Holocene epochs (*42*). Regionally, an oxygen isotope record from Diablo Cave (CBD-2) in Guerrero, Mexico suggests a shift toward drier conditions during the middle Purron phase following several wet centuries at the end of the Abejas phase, with a return to wetter conditions by the middle of the Ajalpan phase (Fig. 6B) (*50*). Although faunal remains from the Purron and Ajalpan phases are limited, results from the few rabbit specimens available for analysis demonstrate relatively high δ^18^O_ap_ and δ^13^C_ap_ values, and the results of a Bayesian mixing model suggest a drier environment. In contrast, deer from the Ajalpan phase exhibit lower δ^18^O_ap_ values, suggesting greater moisture and highlighting taxon-specific ecological responses. Archaeological survey data further indicate that the Purron phase was characterized by low population density and few occupied sites, possibly in response to unfavorable environmental conditions (*79*). Maize cultivation, however, continued during the Purron phase as evidenced by directly dated maize specimens from El Riego and San Marcos caves (*19*, *24*). This phase was also the time in which people first began to make and use ceramic vessels (*27*). In comparison, the subsequent Santa Maria phase (2800 to 2250 cal BP) —marked by rapid population growth, the emergence of town-sized settlements with monumental architecture, and increasing social complexity —corresponds with isotopic evidence for exceptionally wet and forested conditions in the Tehuacan Valley.

Recently, Rosenswig [(*11*) pages 463 to 464; (*80*)] argued that the ultimate cause of the Archaic-Formative period transition and the expansion of sedentary farming in Mesoamerica was the climatic disturbance of ~4200 to 3800 cal BP. His model posits that a multicentury drought incentivized semi-sedentary mixed-strategy horticulturalists to cultivate maize more intensively and exert greater selective pressure on alleles for higher yields, practices that preadapted these communities and their maize populations for rapid demographic and agricultural expansion when wetter conditions returned [e.g., (*11*, *81*)]. The faunal isotope data from the Tehuacan Valley are limited and mixed for this period. Nonetheless, the rabbit data, particularly as expressed through the Bayesian mixing model, provide tentative support to this notion, hinting that dry conditions were present through the Purron and Ajalpan phases. In such a context, domesticates such as maize may have been subjected to greater selective pressure for productivity as human populations increasingly relied on resources that were capable of intensification, especially through canal irrigation. The foraging-to-farming transition was likely a slow, multigenerational process that unfolded over several centuries as maize productivity gradually increased enough to support sedentary multifamily communities. While aridity during the Purron and Ajalpan phases may have catalyzed this process and set the stage for rapid growth during the subsequent Santa Maria phase, additional paleoclimatic and paleoenvironmental data are needed to evaluate this model fully.

A limitation of the study was the small sample sizes from certain phases, which precluded more detailed analyses of environmental change through time. The abundance of faunal bones at archaeological sites is tied to human activity and population numbers, and thus during periods of low human population, less bone material is available. This was the case for the Purron and Ajalpan phases, which were key periods of social change as hunter-gatherers became agriculturalists.

The Tehuacan Valley is important because it represents one of few locations where botanical and archaeological preservation have permitted multiple methodological perspectives, including morphological studies of ancient plant remains, genetics, stable isotope analysis, zooarchaeology, and archaeology, to address long term dynamics of human-plant and human-animal interactions. Results of this multi-isotope analysis of mammal bones from archaeological deposits provide new information on the characteristics of the paleoenvironment and allow us to explore long-term trends in environmental change in tandem with a well-defined record of social change. While climatic or environmental factors may serve as partial and macroscale explanations for the adoption of agriculture, future archaeological research should build on the environmental context established in this study and focus on illuminating the proximate (social, cultural, ecological, and economic) reasons why communities within the valley reduced their seasonal mobility and began sedentary lifestyles focused on intensive maize agriculture.

## MATERIALS AND METHODS

### Chronological placement of samples

Herbivore bone specimens were assigned to one of the nine recognized pre-Colonial chronological phases for the Tehuacan Valley. These assignments were made by a combination of direct radiocarbon dating of specimens and by relative dating through assessments of associated material culture. To verify the chronological sequence used to investigate changes in isotopic values over time, we developed a Bayesian radiocarbon model for the Tehuacan Valley using 94 previously published radiocarbon ages (*23*, *38*, *40*, *82*), including 14 directly dated bone samples included in the present study and one new radiocarbon date on a canine bone (data S1 and table S1). The Bayesian model was constructed in the online version of OxCal 4.4 (*83*) using the IntCal20 calibration curve (*84*) and a CQL2 script. Notably, our revised chronology exhibits several key differences from the traditional chronological sequence of the valley (*38*). Additional information about the Bayesian radiocarbon modeling can be found in the Supplementary Text and in data S1.

### Bone specimens

All bone specimens were originally excavated as part of the Tehuacan Archaeology and Botanical Project directed by MacNeish (*39*) and analyzed by Flannery (*66*). Specimens were stored at the Laboratorio de Arqueozoología of the Instituto Nacional de Antropología e Historia (INAH) in Mexico City. Specimens were analyzed with the permission for destructive analysis granted by INAH and the Consejo de Arqueología (Oficio 401.1S.3-2018/129). In total, the sampled specimens originated from 10 archaeological sites within the valley (table S1) and span the entire occupational sequence (data S2). Adult specimens were prioritized for analysis, as determined by epiphyseal closure. To avoid duplicating samples from the same individual, specific, sided bone elements, such as left mandibles, were sampled from each archaeological level and unit when possible. In total, 79 deer and 101 rabbit bones were included in the analysis (data S2 and table S1). Before destructive analysis, all established metric measurements of bones were taken with a digital caliper following standardized measurements (*85*).

### Sample processing

Preparation of bone samples occurred at the Laboratorio de Isótopos Estables, Laboratorio Nacional de Geoquímica y Mineralogía, Instituto de Geología of the Universidad Nacional Autónoma de México. Each selected faunal bone had the surface cleaned by ablating it with a Dremel handheld rotating saw. All samples were then placed in beakers with ultrapure water and subjected to ultrasonic baths until clean. Each cleaned sample was then partitioned into two subsamples: One in which the bioapatite was extracted to analyze stable carbon and oxygen isotope values in bone carbonate (δ^13^C_ap_ and δ^18^O_ap_) and a second in which the collagen was extracted and purified for stable carbon and nitrogen isotope analysis (δ^13^C_col_ and δ^15^N_col_). Each bone thus yielded four lines of stable isotope data, where preservation permitted.

For bioapatite analysis, powder samples were removed from each bone fragment by a diamond-tipped engraving bit attached to a Dremel rotating saw and were sieved through a fine screen to ensure homogenous particle size. Following procedures based on those of Koch *et al.* (*86*), samples were treated for 48 hours with 2.5% reagent grade sodium hypochlorite (NaOCl), rinsed four times with ultrapure water, and then treated for 24 hours in 0.1 M acetic acid, rinsed four times with ultrapure water, soaked in ethanol, and then dried for at least 24 hours at 90°C in a laboratory oven. Treated samples were analyzed on a Gas Bench II with a GC PAL auto-sampler connected to a Thermo-Finnigan MAT 253 mass spectrometer through a ConFlo IV interface. Powdered samples were reacted with 100% phosphoric acid to liberate CO_2_. Resulting δ^18^O and δ^13^C values were normalized to the V-PDB scale with NBS-19 (*87*). The accuracy of the analysis was checked using an internal calcite reference analyzed every seven samples. For this technique both the accuracy and precision were 0.2‰ for oxygen and 0.2‰ for carbon. An internal rabbit bone standard that was analyzed in triplicate resulted in precision and accuracy of δ^13^C = −15.8 ± 0.1‰ (expected −15.8‰ ± 0.1) and δ^18^O = −4.9 ± 0.2‰ (expected −5.0 ± 0.2‰).

For collagen analysis, sample preparation followed procedures similar to the “chunk” method described by Sealy *et al.* (*88*). Small pieces (~2 mm diameter) were decalcified for approximately 2 weeks in 0.25 M hydrochloric acid (HCl) at room temperature, changing the HCl solution every 48 to 72 hours until complete demineralization. After rinsing to neutrality, humic acids were removed by soaking samples for 6 hours in 0.125 M sodium hydroxide (NaOH). Samples were then centrifuged and rinsed three times with ultrapure water. Next 0.25 M HCl was added to each sample for 60 min, before they were centrifuged and rinsed three additional times to neutralize the pH. To solubilize the collagen samples, 12 ml of ultrapure water were heated to 85°C and the water was adjusted to pH 3 by adding 2 ml of 0.01 M HCl. The tubes were capped and left in the laboratory oven for 20 hours. The solubilized collagen samples were then filtered through 60- to 90-μm Elkay brand Ezee filters, frozen, and lyophilized at −52°C and at 0.03 mbar for 24 to 48 hours. For stable isotope analysis, a sample of 0.6 mg of purified collagen was weighed in a tin capsule and analyzed using an Organic Elemental Analyzer FLASH 2000 attached via a ConFlo IV interface to the MAT 253 Mass Spectrometer. Pure CO_2_ and N_2_ gases calibrated with Oztech tanks were used as working standards. To normalize the results for δ^13^C, we used the reference materials NBS-22, PEF1, IAEA CH6, and every 10 samples two internal laboratory references glycine and l-serine (Sigma-Aldrich) were also analyzed. To normalize δ^15^N values, reference materials IAEAN1, USGS25, USGS26, USGS 40, and USGS 41 were used; the internal reproducibility for δ^13^C was ±0.03‰, and for δ^15^N was ±0.01‰.

### Diagenesis

To assess the degree of preservation of skeletal tissues and to eliminate those that may not preserve biogenic stable isotope values, we used several measures of diagenesis. For apatite, we used Fourier Transform Infrared Spectroscopy with the attenuated total reflection technique (Thermo Fisher Scientific Nicolet iS10 using a GladiATR accessory with a diamond crystal). We assessed the infrared splitting factor (IR-SF) and the ratio of bone carbonate to phosphate (C/P) (*89*, *90*). The IR-SF indicates bone crystallinity; higher values suggest recrystallization due to diagenesis. The C/P ratio reflects chemical composition changes, often altered by postmortem processes. Significant shifts in either metric from expected values indicate structural or chemical degradation (*89*). On the basis of previous literature (*83*) and internal laboratory studies, we set the acceptable range for IR-SF between 2.5 and 4.0, and the acceptable range of C:P between 0.10 and 0.35. Samples that fell outside of acceptable ranges were not considered in the interpretation of the results. For collagen preservation, we assessed the atomic ratios of carbon to nitrogen (C:N) with acceptable values from 2.9 to 3.6 (*84*).

The results of the Fourier transform infrared–attenuated total reflectance analysis found that all bioapatite specimens yielded acceptable confidence interval values but that two specimens (ADS-0112 and ADS-0116) exhibited C:P values below the 0.10 cutoff. Two additional bioapatite samples (ADS-0095 and ADS-174) were lost during sample preparation. Many fewer collagen specimens than bioapatite specimens yielded acceptable data. Of the 180 total specimens, only 100 yielded sufficient collagen after the processing and purification steps. Of these, an additional 19 exhibited C:N ratios higher than the 3.6 cutoff, suggesting diagenetic alteration of the samples.

### Comparative paleoclimate data

To contextualize the Tehuacan stable isotope data from faunal bone specimens within a broader climatic context, we accessed high-resolution paleoclimate records using the Paleo Data Search tool (https://ncei.noaa.gov/access/paleo-search/) (*87*) hosted by the National Centers for Environmental Information (https://ncei.noaa.gov/).

### Statistical analysis

All statistical analyses and visualization of the data were done in the R computing environment using R version 4.2.3 (*91*). Parametric statistical analyses were used to test differences in stable isotope values between the geological subepochs, and cultural phases. Preliminary Levene’s tests for homogeneity of variance indicated that variances differed significantly between rabbits and deer for δ^13^C_ap_, δ^18^O_ap_, and δ^13^C_col_ values (all *P* < 0.05), but not for δ^15^N_col_ values (*P* = 0.57). Shapiro-Wilk tests showed that δ^13^C_ap_ and δ^15^N_col_ values were normally distributed for both taxa, whereas δ^18^O_ap_ in deer and δ^13^C_col_ in rabbits exhibited mild departures from normality. Because Welch’s *t* test is robust to moderate variance in heterogeneity and minor deviations from normality, and to maintain a consistent and conservative analytical framework, we used Welch’s unequal-variance *t* tests, which automatically adjust the degrees of freedom, for all pairwise comparisons between isotope values of the two genera. For comparisons of more than two groups, we used ANOVA tests. Tests for normality and homogeneity of variances indicated that three of the four isotope variables met ANOVA assumptions, while δ^18^O_ap_ showed a mild deviation (Levene’s test *P* = 0.048). Given the robustness of ANOVA to moderate variance heterogeneity and the similar group sizes in our dataset, we applied standard one-way ANOVA with Tukey’s HSD post hoc tests for all isotope variables between subepochs. For comparisons of isotope values between eight different cultural phases, we used Welch’s ANOVA tests, which do not assume equal variances and are considered to be more robust than the standard ANOVA when equal variances are not assumed or when sample sizes differ. Significance levels were set at α = 0.05 for all models.

To assess the potential influence of geographic location of archaeological site on measured stable isotope values in faunal bone, we ran generalized additive models (GAMs), assuming gaussian responses, to fit the isotope variables (four separate models) as functions of latitude, longitude, and elevation (see link for code). Results for individual GAMs revealed weak fits for spatial coordinates with stable isotopes (*R*^2^ range from 0.2 to 0.005). The only significant predictors were longitude for δ^18^O_ap_ values on both taxa, and elevation for δ^13^C_ap_ on rabbits. Considering only rabbits, there was a significant positive relationship between elevation and δ^13^C_ap_ values, but not latitude or longitude and δ^13^C_ap_ values. This trend, however, is largely driven by the high δ^13^C_ap_ values from specimens from the Pleistocene contexts of Coxcatlan Cave, which is the highest elevation site, but also the site with 95% of the Pleistocene rabbit specimens (18 of 19). As the fauna from Pleistocene contexts of the cave lived during an epoch characterized by drier conditions and a grassier landscape (discussed more above), we believe that the significant relationship between δ^13^C_ap_ values and elevation is due to temporal changes in the environmental landscape rather than geographic position of the site. This trend also likely explains the significant relationship between oxygen and longitude as Coxcatlan is one of the easternmost sites of the sample. For deer, no significant relationships were found between space and both carbon and oxygen stable isotope values. The sample sizes for isotope values from collagen were too small to run GAMs. We compared the general trends we observed over time in the valley-wide sample with the trends within the singular site of Coxcatlan Cave, which had the largest sample size, finding similar patterns at the regional and site-level scales (fig. S3). On the basis of the results, we believe that it is acceptable to combine data from multiple sites to explore the general paleoenvironment in the valley through time.

### Stable isotope mixing model

We use the Bayesian stable isotope mixing package, MixSIAR (*92*) through the R computing environment (*91*) to interpret the isotope variables in terms of paleoenvironmental changes over time. The model assumes idealized isotopic signatures of three general environments (desert, grassland, and forest) and assesses how stable isotope values from faunal bones within the Tehuacan Valley reflect changes in the composition of these patches over time. We focus exclusively on rabbit bone specimens because they are more abundant than deer within our sample and because comparative data exist on rabbit stable isotope values from across Mexico (*37*, *43*). We base expected values on rabbit values from similar environmental conditions and on known relationships between environmental conditions and isotopic values in mammals (*37*, *43*, *93*, *94*). For the desert environment, we assume rabbit bones from these environments will exhibit high oxygen and nitrogen stable isotope values and moderate to low stable carbon isotope values due to the lack of C4 grasses. We modeled expected values on archaeological specimens from the Sonoran desert in southern Arizona, USA, an environment with a high prevalence of columnar cacti, shrubs, and low rainfall, similar to large areas of the Tehuacan Valley today [(*43*), page 105063]. For grasslands, we assume that the C4 grasses of the region would result in high stable carbon isotope values, and the open landscape would result in moderately high stable oxygen and nitrogen isotope values. We based our values on leporid specimens from the grasslands of the Central Mexican Matorral desert of modern Zacatecas, Mexico (*43*), but we increase the stable carbon isotope values of the model relative to previously published values (δ^13^C_ap_: from −4.9 to −2.0‰; δ^13^C_col_: from −11.5 to −9‰) to represent an idealized version of the C4-grassland patch. To model a forested landscape, we based the values after leporids from the slopes of the Sierra Madre Occidentals on the ecotone with the Chihuahuan desert (*43*), but we lower the stable carbon isotope values (δ^13^C_ap_: from −6.1 to −13.0‰; δ^13^C_col_: from −16.1 to −20‰) to highlight C3 plants of the forest patch. Mean and SDs of the isotope values that are representative of these idealized environmental types are provided in table S6 and visualized in fig. S4. Posterior results of the model are presented in table S7 and Fig. 4.

For the MixSIAR model, no trophic offset was used as the idealized environmental patches are already expressed in values equivalent to leporid bone isotope values. We use an uninformed prior for each environmental source. Three Markov Monte Carlo chains were run with the “normal” argument where chain length was set as 100,000, and the burn was set at 50,000. Chains were thinned at 50. We set the model to account for “residual error” but not for “process error.” To ensure chain convergence, we assessed the results of Gelman-Rubin and the Geweke Diagnostics.

## Supplementary Materials

### The PDF file includes:

Supplementary Text Figs. S1 to S4 Tables S1 to S7 Legends for data S1 and S2 References
