# Supplementary material for: Multi-isotope analysis of mammal bones provides environmental context for the adoption of agriculture in the Tehuacan Valley of Mexico
Source: Sci Adv. 2026 Mar 4;12(10):eadw9222. doi: 10.1126/sciadv.adw9222 (PMC12959416; doi:10.1126/sciadv.adw9222)
Supplement: Supplementary file 1 — Supplementary Text Figs. S1 to S4 Tables S1 to S7 Legends for data S1 and S2 References [file sciadv.adw9222_sm.pdf]

Science Advances
AAAS

Supplementary Materials for

Multi-isotope analysis of mammal bones provides environmental context for the adoption of agriculture in the Tehuacan Valley of Mexico

Andrew D. Somerville *et al.*

Corresponding author: Andrew D. Somerville, asomervi@iastate.edu

Sci. Adv. **12**, eadw9222 (2026)
DOI: 10.1126/sciadv.adw9222

The PDF file includes:

Supplementary Text
Figs. S1 to S4
Tables S1 to S7
Legends for data S1 and S2
References

Other Supplementary Material for this manuscript includes the following:

Data S1 and S2

Supplementary Text

Bayesian Radiocarbon Model

To situate the stable isotope data from the faunal bone samples within a temporal framework, we build on previous assessments of the chronology of the Tehuacan Valley (*20*, *38*, *40*, *78*) and develop a Bayesian radiocarbon model for the cultural phases identified by the original excavators. We compile a database of previously produced radiocarbon ages that have associated stratigraphic and cultural information. Most samples were obtained from the early radiometric analyses of samples by Johnson and Macneish (*38*), but additional AMS radiocarbon ages were obtained on plant samples (*20*, *23*, *83*) and on a sample of faunal bones included in the present study (*40*). In addition, we report the results of one new AMS radiocarbon assay on a canine bone from Coxcatlan Cave (UCIAMS-223935, ADS-0014, 2725 ± 15 BP; See Data S1). Note that, after reassessments of the directly dated bone specimens, we have changed three of the faunal IDs of Ajuereado phase rabbits that were misidentified as *Lepus* sp. in a previous publication (*40*) to *Sylvilagus cunicularius* (see Data S1). In total, we compiled 168 radiocarbon samples from the Tehuacan Valley including bone samples, plant tissues, wood, cloth, charcoal, and mixed material samples (Fig. S1). While additional radiocarbon analyses have been conducted in the valley outside of our selected dataset (*19*, *24*, *25*, *76*), these specimens do not have comparable stratigraphic and cultural information to the specimens recovered and analyzed from the original project and we thus do not include them in the Bayesian model.

Within the sample of 168 selected radiocarbon ages, we manually reject samples that are out of sequence for the depositional history of the site, likely due to human and rodent activities of digging and disturbing existing floors. We identify two types of outliers: those that were inconsistent with their specific stratigraphic zone (slightly out of sequence), and those that were inconsistent with the broader cultural phase to which they were assigned (significantly out of sequence). In total, we excluded 74 of the 168 specimens (44%) from the Bayesian model (Table S1). While this number of excluded specimens is high, different materials had differential probabilities of matching their stratigraphic/cultural phase or zone. Most plant samples (72.5%) were rejected because of the clear mismatches between their stratigraphic placement and the measured radiocarbon age. In contrast, however, only three (20%) bone samples were rejected from the model. Moreover, these three bones were only slightly out of place for their zone. Fifteen out of fifteen bone samples (100%) correctly matched their predicted cultural phase. We therefore have a high degree of confidence that the bones of the present study, which included the 14 directly dated faunal bone specimens, accurately represent the broad chronological phase to which we assign them in this study.

The Bayesian model was constructed in the online version of OxCal 4.4 (*96*) using the IntCal20 calibration curve (*97*) and a CQL2 script as a sequence of 10 cultural phases within the valley. The Santa Maria, Palo Blanco, and Venta Salada phases, however, were split into Early and Late components, making 13 total phases. Most phases were composed of specimens from multiple sites. For example, the Coxcatlan phase was represented by radiocarbon specimens from Coxcatlan Cave, Purron Cave, and San Marcos Cave. In cases where a site had several stratigraphic zones (levels) that correspond to a single cultural phase, we included the radiocarbon samples in a *sequence* within the broader cultural *phase* (Data S1). After final

sample selection, one radiocarbon specimen (I-929) returned an agreement index of 50.3%, which is less than the recommended 60% cutoff. However, this specimen is the only radiocarbon sample from Zone H of the Ajalpan site, and its age distribution appears in congruence with the contexts above and below it in the stratigraphic sequence, and we therefore leave it in the model. After running the final model, overall agreement indices indicate that the prior model highly agreed with the observations: $A_{model} = 160.1$, $A_{overall} = 160.3$.

Overall, our modeling of radiocarbon ages across multiple sites within the valley corresponds well with initial assessments of the chronology and with more recent assessments of the sequence of Coxcatlan Cave (*40*, *78*). However, our revised chronology of the valley has multiple areas of difference with the original sequence as presented by Johnson and MacNeish (*38*). While the Johnson and MacNeish chronology suggested the range for the El Riego period was 9350-7750 cal BP, our results suggest that it spanned 9900-7900 cal BP, which is range that begins 550 years earlier and ends 150 years earlier. Similarly, our model for the Coxcatlan phase (7900-6300 cal BP) begins 150 years earlier and ends 200 years earlier than the traditional estimate (7750-6100 cal BP). Notably, our model lengthens the duration of the Abejas phase (6300-4550 cal BP), which is the period in which maize first arrived in the Tehuacan valley. Our model estimates that the Abejas phase began 200 years earlier and ended 250 years later than the traditional estimate (6100-4800 cal BP). Relative to the Johnson and MacNeish chronology, our model pushes back the beginning and end of the Purron phase, which is the first phase of the Formative period. Our model estimates that the Purron phase dates to 4550-3250 cal BP, which is a range that begins 250 years later and ends 600 years later than the traditional model (4800-3850 cal BP). Our estimate for the Ajalpan phase (3250-2800 cal BP) begins 600 years later and ends 150 years later than the Johnson and MacNeish estimate (3850-2950 cal BP). Our model estimates that the Santa Maria phase (2800-2250 cal BP) begins 150 years later and ends 150 years earlier than the traditional chronology (2950-2100 cal BP). For the Palo Blanco phase, our estimated range of (2250-1250 cal BP) begins 150 years earlier and ends 80 years earlier than the earlier estimate (2100-1170 cal BP). Finally, our model estimates that the Venta Salada phase (1250-429 calBP) began 80 years earlier than the estimated range of Johnson and MacNeish. The Venta Salada phase and the overall chronological sequence end with the arrival of the Spanish at 429 cal BP (AD 1521). This revision of the chronological sequence is important as it allows for better comparisons with the archaeological and paleoenvironmental record of the Tehuacan valley with relevant records from other regions.

Isotope Ecology of Rabbits and Deer

Isotopic values obtained from mammalian bones can to serve as archives of paleoenvironmental information and reflect the environmental context in which past humans lived (*32*, *34*, *98*). This study focused on bone samples from deer (*Odocoileus virginianus*) and rabbits (*Sylvilagus* spp.) due to their abundance, their generalized diets, their relatively small home ranges, and the length of their lifespans. Because rabbits and deer were the most commonly encountered fauna during excavations, they were available for sampling across all chronological phases (*66*).

Deer specimens were identified as *Odocoileus virginianus*, which is common to the region of southern Puebla, Mexico. For the rabbits, two distinct morphotypes were observed, a large and small variety. The large specimens were initially identified as *Sylvilagus cunicularius* (Mexican

cottontail or mountain rabbit) and the small specimens were identified as *Sylvilagus audubonii* (desert cottontail) (*66*). Subsequent to the early classification, however, later studies by Flannery, cast doubt on the identifications and in the ability to correctly identify cottontails to specific species without the complete skeletons (*99*:53). In particular, *S. audubonii* is skeletally indistinguishable from *S. floridanus connectens* except for the morphology of the tympanic bullae, which are rarely preserved in the archaeological record. Seventeen species of cottontail rabbits exist in Mexico, and the species with the widest distribution, *Sylvilagus floridanus*, has 30 described subspecies (*42*:138). This diversity of leporids makes precise taxonomic classification by morphology exceedingly difficult in Mesoamerica. For this study then, we assume the larger specimens are *S. cunicularius* but we identify the smaller varieties only as "small" cottontails, with the understanding that they likely represent either *S. floridanus connectens* or *S. audubonii*. Across its range, *S. cunicularius* is sympatric with multiple rabbit species, including *S. floridanus connectens* or *S. audubonii*, due to its habitat tolerance for grasslands, shrublands, and pine-oak forests (*42*). Like the smaller central Mexican leporids, the Mexican cottontail is a generalist herbivore consuming grasses, forbs, and shrubs, but it has a larger habitat tolerance than smaller cottontails, inhabiting both mountain forests and grassland patches. In the Tehuacan Valley the greater tolerance for grasses and open habitats by *S. cunicularius* may have led it to exploit the grassland patches of the valley more than the smaller cottontails, which prefer habitats with more scrub vegetation for cover (*66*:147).

Both rabbits and deer are herbivorous browsers that consume a wide variety of plants within their habitats (e.g., *100*, *101*), which causes their isotopic values to represent an averaged input of the botanical composition of the local landscape. The size of the home-ranges of white-tailed deer depend on multiple factors, including individual age and sex and on environmental factors such as vegetative cover and habitat diversity, but their ranges typically vary between approximately 60-520 ha (*102*, *103*:6). Rabbits tend to have smaller home-ranges, typically under 5 ha (*104–106*). Both rabbits and deer have relatively short lifespans. Rabbits rarely live more than three years and usually less than one (*107*). While white tailed deer can live up to 20 years, they usually only live between two to five years, with females typically living longer than males (*103*). Both taxa generally live long enough for the individuals to have experienced the environmental extremes of the wet and dry seasons and hence have integrated the isotopic values of plants during these periods. The qualities of generalized herbivorous diets, small home-ranges, and short lifespans, in addition to their archaeological abundance, make leporids and deer well-suited to reflect key properties of the environmental landscape through the isotopic values ($\delta^{13}C$, $\delta^{18}O$, $\delta^{15}N$) of their skeletal tissues.

<u>Stable Carbon Isotope Analysis</u>

The $\delta^{13}C$ of herbivorous animal bone tissue are influenced by the $\delta^{13}C$ values of consumed plants (*29*, *108*). Plants derive carbon from atmospheric CO_2, which is fixed in plant tissues through photosynthesis. Plant $\delta^{13}C$ values vary significantly across different photosynthetic pathways (*109*, *110*). Most plants utilize the C_3 (Calvin-Benson) photosynthetic pathway and exhibit $\delta^{13}C$ values from −35 to −20 ‰ (*111*, *112*). Plants utilizing the C_4 (Hatch-Slack) photosynthetic pathway are primarily dry-adapted grasses, such as millet and maize, and exhibit $\delta^{13}C$ values ranging from −15 to −7 ‰ (*112*). Crassulacean acid metabolism (CAM) photosynthesis is utilized by cacti and succulents, which have $\delta^{13}C$ values that overlap substantially with C_4 plants

(*110*, *112*). While C_3 plants are found in most moderate and temperate regions, C_4 and CAM plants occur in environments characterized by low moisture, warm temperatures, and long periods of sunlight (*113*). Because of these differences, stable carbon isotope values from herbivore bone, which incorporates can serve as general proxies for the botanical composition of past landscapes.

Local environmental factors can also influence ^{13}C abundance in C_3 plants, causing them to range an additional 3-6 ‰ (*111*, *114*). Water stress causes greater stomatal closure in C3 plants and decreases ^{13}C discrimination during photosynthesis, which leads to elevated $^{13}C/^{12}C$ ratios in plant tissues (*114*, *115*). High soil salinity can also elicit stomatal closure and decreased ^{13}C discrimination in C3 plants, leading to elevated $\delta^{13}C$ values (*115*). Finally, plants from closed canopy forests exhibit lower $\delta^{13}C$ values than plants from open habitats, a trend caused by less solar irradiance in forest understories (*116*) and plant recycling of ^{13}C-depleted CO_2 in denser forested environments (*117*, *118*). Therefore, important for paleoenvironmental reconstruction is the pattern that herbivores feeding in warmer, drier, and more open environments exhibit higher $\delta^{13}C$ values than herbivores foraging within cooler, wetter, and more closed environments. Stable carbon isotope values are obtained from both bone mineral apatite and from organic bone collagen. $\delta^{13}C$ data from these tissues, however, reflect different dietary inputs. $\delta^{13}C$ values from bone collagen ($\delta^{13}C_{col}$) are heavily biased towards dietary protein while $\delta^{13}C$ values from bone apatite ($\delta^{13}C_{apatite}$) reflect $\delta^{13}C$ values from the whole diet (*119–121*). Analyzing both tissues, then, provides a more complete picture of the sources of dietary carbon and a more nuanced picture of the past landscape in which the animals were feeding.

Stable Oxygen Isotope Analysis

For homeothermic mammals, $\delta^{18}O$ values from bone mineral apatite ($\delta^{18}O_{ap}$) are in equilibrium with $\delta^{18}O$ values of body water (*122*, *123*), which is determined by the $\delta^{18}O$ values of ingested water (*31*, *124*), whether imbibed or consumed as plant material. The abundance of ^{18}O relative to ^{16}O in meteoric water, the primary source of drinking water, depends upon a number of environmental factors, including elevation, distance from the sea, the amount of rainfall, relative humidity, and temperature (*125–127*). Variation in oxygen isotope values in plant leaf water is influenced by evaporative enrichment of ^{18}O, and varies as a factor of relative humidity and precipitation (*37*, *128–130*).

Stable oxygen isotope values in bone mineral can be measured in both the structural carbonate (CO_3^{2-}) and phosphate (PO_4^{3-}) components of the hydroxyapatite (generalized as $Ca_{10}[PO_4, CO_3]_6[OH, CO_3]_2$). This study focused on oxygen isotope values from structural carbonate within the bone apatite ($\delta^{18}O_{ap}$). For herbivore mammals within a given region, variation in $\delta^{18}O$ values depends on changes in variables such as rainfall, humidity, and temperature (*34*). Herbivores, such as rabbits and deer, that obtain their body water primarily through consumed vegetal material exhibit $\delta^{18}O_{ap}$ values that correlate negatively with relative humidity and mean annual precipitation (*43*, *128*, *129*).

Stable Isotopes of Nitrogen

The $\delta^{15}N_{col}$ values of organic tissues of herbivore are primarily influenced by the $\delta^{15}N$ values of the plants they consume (*30*, *108*). Primary sources of N for plants are inorganic soil nitrate (NO_3^-) and ammonium (NH_4) (*131*). Aside from leguminous plants, which exhibit $\delta^{15}N$ values close to 0 ‰ due to their symbiotic relationship with soil bacteria that fix N_2 from atmospheric nitrogen (*132*), soil and plant $\delta^{15}N$ values globally correlate negatively with mean annual precipitation (MAP) and positively with mean annual temperature (*36*, *133–135*). Warm and dry environments tend to be open systems and are prone to soil N isotope fractionation processes that lead to enrichment of ^{15}N in soil substrates, including mineral leaching, denitrification, NH_3 volatilization, and increased microbial activities (*134*). Cool and wet closed systems conserve and recycle mineral N more efficiently and have less microbial activity, leading to lower $\delta^{15}N$ values (*133*, *136*, *137*).

Like the plants that they consume, animals also exhibit higher $\delta^{15}N_{col}$ values in hot and dry environments relative to cooler and wetter ones, presumably due to the relationships between plant $\delta^{15}N$ values and moisture and temperature variability (*32*, *33*, *138*, *139*). Environmental variables at more localized levels, such as local topography and soil salinity, also influence soil and plant and hence animal $\delta^{15}N$ values (*94*, *140*). Because of the effects that temperature and humidity can have on soil and plant $\delta^{15}N$ values, the $\delta^{15}N_{col}$ values from mammalian skeletal tissue can be used as a general proxy for past environmental conditions.

Provenance

All deer and rabbit bone specimens of the present study were recovered from archaeological excavations at 10 sites (Table S2) within the Tehuacan Valley of southern Puebla, Mexico during four seasons of research (1961–1964) by the Tehuacan Archaeological-Botanical Project led by Richard S. MacNeish (*80*). Since their excavation, cleaning, and initial analysis, the bones of the Tehuacan faunal collection have been stored at the Laboratorio de Arqueozoología, Subdirección de Laboratorios y Apoyo Académico, Instituto Nacional de Antropología e Historia, in Mexico City, Mexico, directed by JAC.

Species identifications were made by KF in the early 1960s with the aid a comparative collection from the area (*66*). Sample selection for stable isotope analysis occurred in 2017-2018 by ADS, at which time species identifications, as labeled on the bags, were confirmed by ADS and JAC, using a comparative collection when necessary. Each bone specimen was assigned to a chronological phase based on stratigraphic placement and Bayesian modeling of available radiocarbon dates, including 14 directly dated bone specimens used in this study (Table S1; Data S1).

Destructive analysis of bone specimens occurred with permission of the Consejo de Arqueología (Oficio 401.1S.3-2018/129). Preparation and analysis of samples occurred in the Stable Isotope Laboratory at the Universidad Nacional Autónoma de México in Mexico City. Sample preparation was done by RHF, ADS, FO, ECA, and IC, stable isotope analysis was performed by FO and ECA, and the laboratory was directed by PM and LBO. Data analysis and interpretation were primarily performed by ADS, IC, LBO, and DD. In cases where the entire sample was not consumed by stable isotope analysis, remaining bone samples were returned to the larger

collection of animal bones from the Tehuacan Valley in the Laboratorio de Arqueozoología in Mexico City where they remain under the curation of JAC.

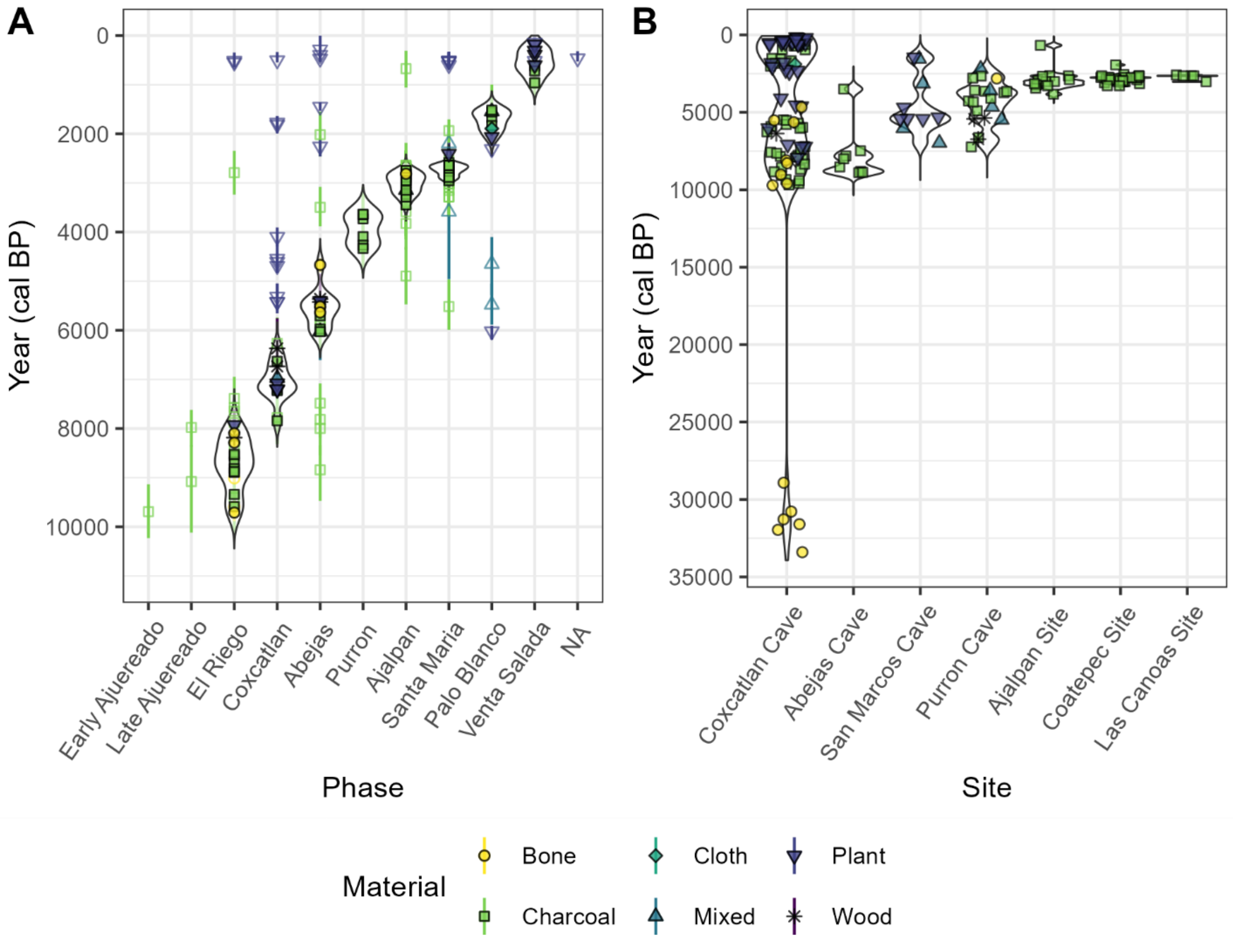

Fig. S1.

Violin and strip plots of calibrated radiocarbon ages from archaeological sites within the Tehuacan Valley. Markers represent median values and error bars represent the 2 SD (95.4 %) range. (**A**) Radiocarbon ages of all Holocene specimens grouped according to cultural phase assignment based on their stratigraphic context and associated cultural artifacts. Filled symbols represent samples used to construct the Bayesian chronological model. Open symbols represent samples that were excluded from the model because they were out of sequence for their stratigraphic zone or cultural phase. Samples older than 12,000 cal BP were excluded from this graph for visualization purposes. (**B**) All radiocarbon ages grouped by archaeological site.

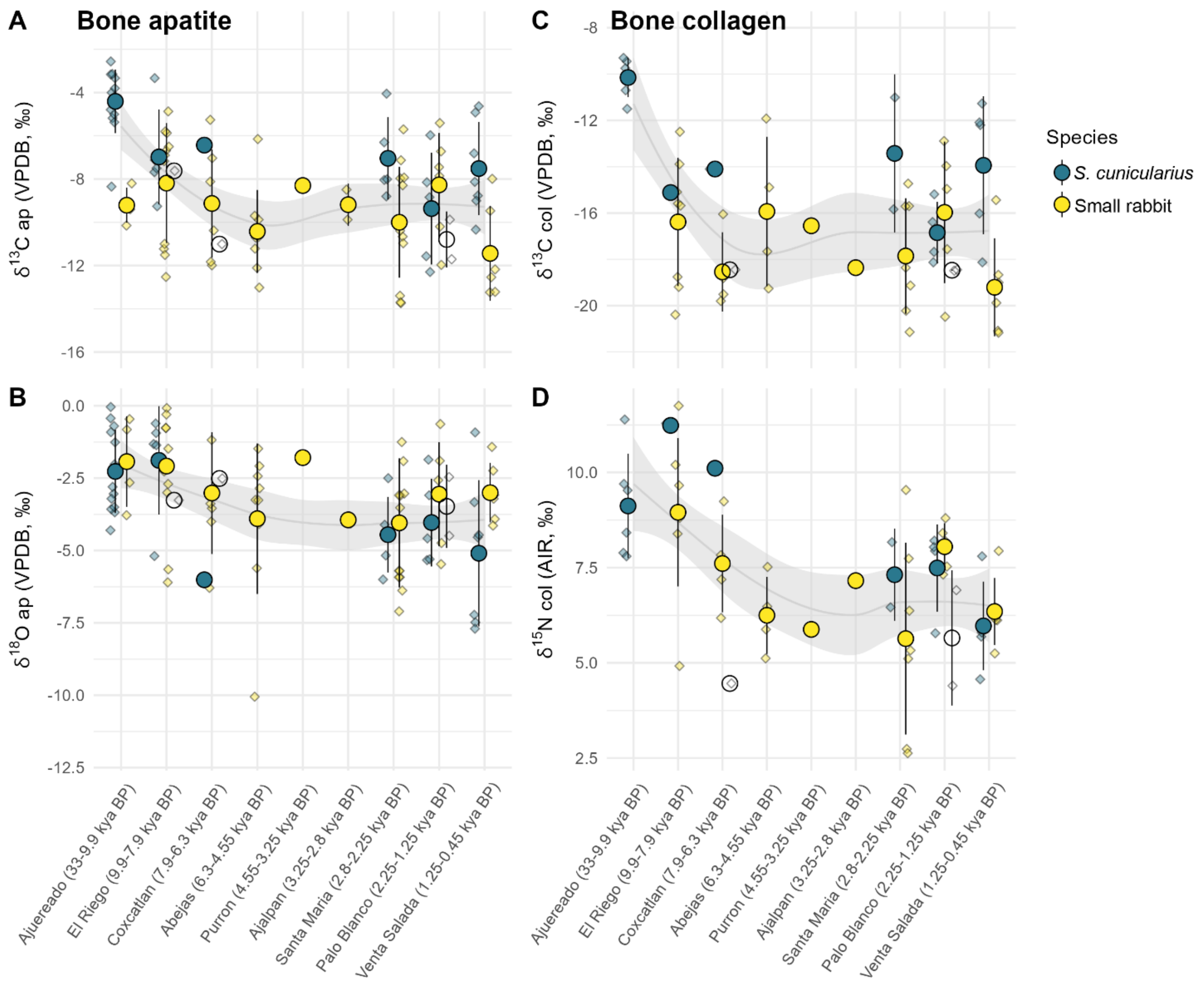

Fig. S2.

Strip plots of stable isotope values of rabbit (*Sylvilagus* spp.) bones from the Tehuacan Valley. Plots are grouped by chronological phase and by rabbit species. Rabbit species are either *Sylvilagus cunicularius* (filled blue markers), small (either *S. audubonii* or *S. floridanus connectens*: filled yellow markers), or unidentified rabbit type (open markers). Large circles represent phase means and small diamonds represent individual bone samples. Error bars denote 1 SD. The shaded gray area around the regression line indicates the 95 % confidence interval band generated with a locally estimated scatterplot smoothing (LOESS) function using the pooled rabbit sample. (**A**) Stable carbon isotope values from bone apatite carbonate. (**B**) Stable oxygen isotope values from bone apatite carbonate. (**C**) Stable carbon isotope values from bone collagen. (**D**) Stable nitrogen isotope values from bone collagen.

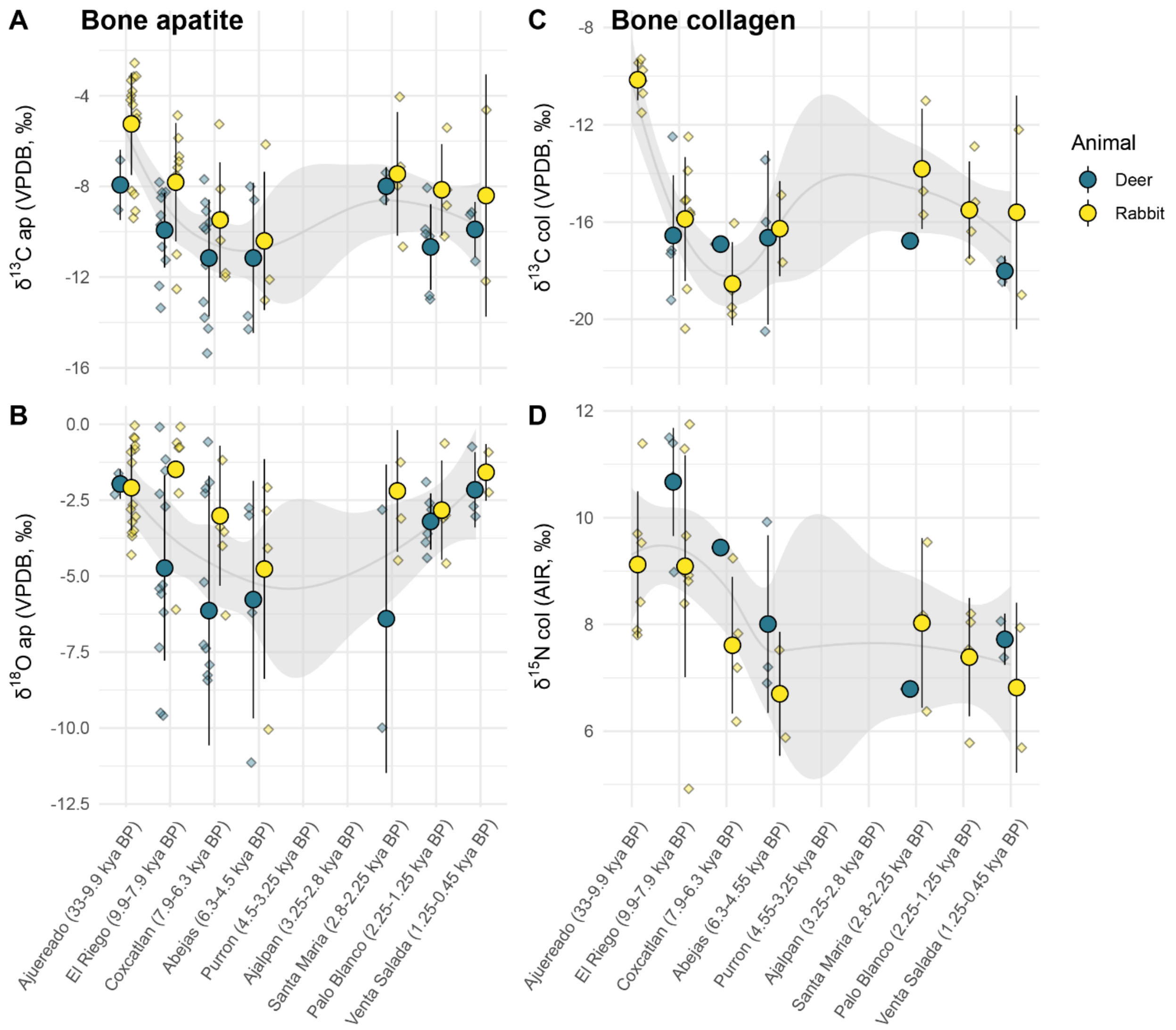

Fig. S3.

Strip plots of stable isotope values of deer and rabbit bones from Coxcatlan Cave. Plots are grouped by animal and chronological phase. Large circles represent phase means and small diamonds represent individual bone samples. Error bars denote 1 SD. The shaded gray area around the regression line indicates the 95 % confidence interval band generated with a locally estimated scatterplot smoothing (LOESS) function using the pooled deer and rabbit sample. (**A**) Stable carbon isotope values from bone apatite carbonate. (**B**) Stable oxygen isotope values from bone apatite carbonate. (**C**) Stable carbon isotope values from bone collagen. (**D**) Stable nitrogen isotope values from bone collagen.

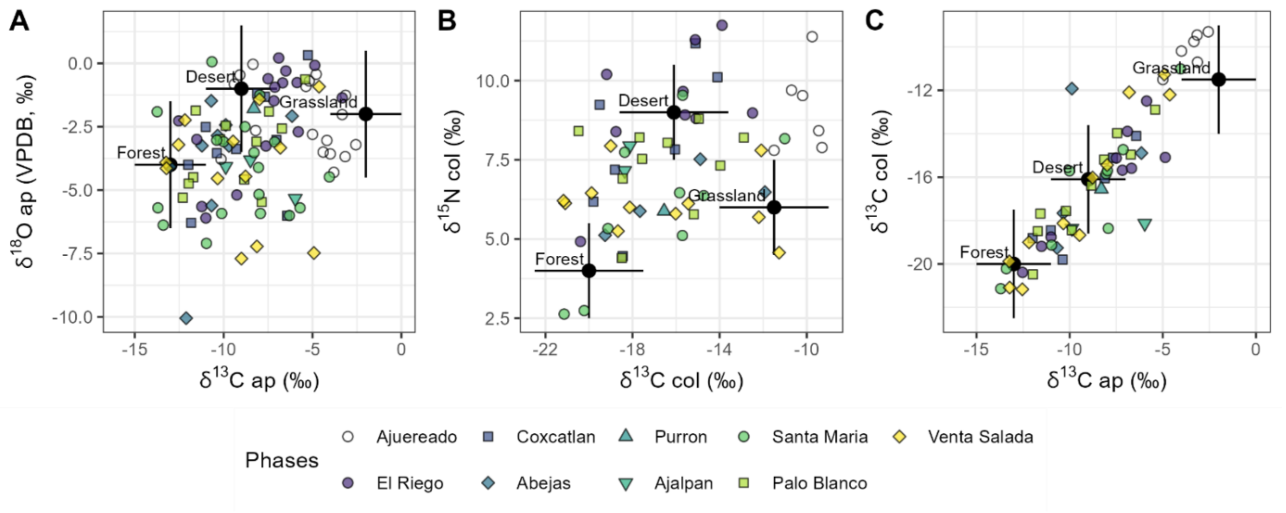

Fig. S4.

Scatterplots displaying the relationship of rabbit stable isotope values form the Tehuacan valley with values of generic environment types of Mexico. Black circles represent environmental type mean values. Error bars represent 1SD from mean of environment type.

Material	Total	Accepted for Radiocarbon Model	Excluded from Radiocarbon Model	Percent Excluded
Bone	15	12	3	20%
Charcoal	99	61	38	38.4%
Cloth	1	1	0	0%
Mixed	8	4	4	50%
Plant	40	11	29	72.5%
Wood	5	5	0	0%
**Total**	**168**	**94**	**74**	**44%**

Table S1.

Counts of radiocarbon samples of different material types that were considered for the Bayesian chronological model of the Tehuacan Valley.

Site Code	Site Name	Odocoileus (n)	Sylvilagus (n)	Total
Tc254	San Marcos Cave	0	6	6
Tc255	Tecorral Cave	0	3	3
Tc272	Purron Cave	5	11	16
Tc307	Abejas Cave	4	5	9
Tc35	El Riego Cave	5	18	23
Tc50	Coxcatlan Cave	43	45	88
Ts204	Ajalpan Site	12	1	13
Ts218	Quachilco Site	0	4	4
Ts367	Ajalpan Locality	0	1	1
Ts368	Coatepec Site	10	7	17
Total		79	101	180

Table S2.

Number of animal bone specimens from each archaeological site sampled for stable isotope analysis.

	$\delta^{13}C$ ap (VPDB, ‰)				$\delta^{18}O$ ap (VPDB, ‰)				$\delta^{13}C$ col (VPDB, ‰)				$\delta^{15}N$ col (AIR, ‰)			
Animal	N	Mean	SD	SE	N	Mean	SD	SE	N	Mean	SD	SE	N	Mean	SD	SE
Deer	99	-9.8	2.2	0.2	99	-5.0	2.9	0.3	59	-17.3	2.4	0.5	59	8.2	2.3	0.5
Rabbit	77	-8.4	2.8	0.3	77	-3.2	2.1	0.2	22	-16.1	3.3	0.4	22	7.4	2.0	0.3
Total	176	-9.1	2.7	0.2	176	-4.1	2.6	0.2	81	-16.7	3.1	0.4	81	7.8	2.1	0.2

Table S3.

Descriptive statistics of stable isotope data from deer and rabbit bones. The table includes the sample size (N), the mean stable isotope values, standard deviation (SD), and standard error (SE).

Phase	Rabbit morphotype	N	$\delta^{13}C_{ap}$ mean (‰)	$\delta^{13}C_{ap}$ SD (‰)	$\delta^{18}O_{ap}$ mean (‰)	$\delta^{18}O_{ap}$ SD (‰)	$\delta^{13}C_{col}$ mean (‰)	$\delta^{13}C_{col}$ SD (‰)	$\delta^{15}N_{col}$ mean (‰)	$\delta^{15}N_{col}$ SD (‰)
Venta Salada	Small	6	-11.4	2.2	-3.0	1.0	-19.2	2.1	6.4	0.9
	Large	7	-7.5	2.1	-5.1	2.5	-13.9	3.0	6.0	1.2
	sp.	0								
Palo Blanco	Small	6	-8.3	2.4	-3.1	1.8	-16.0	3.1	8.1	0.6
	Large	5	-10.2	2.0	-3.7	1.5	-16.4	1.2	7.3	1.4
	sp.	2	-10.8	1.3	-3.5	1.4	-18.5	0.0	5.7	1.8
Santa Maria	Small	13	-10.0	2.6	-4.0	2.2	-17.9	2.5	5.6	2.5
	Large	5	-7.0	1.9	-4.5	1.3	-13.4	3.4	7.3	1.2
	sp.	0								
Ajalpan	Small	2	-9.2		-3.9	0.2	-18.4		7.2	
	Large	0								
	sp.	0								
Purron	Small	1	-8.3		-1.8		-16.6		5.9	
	Large	0								
	sp.	0								
Abejas	Small	9	-10.4	1.9	-3.9	2.6	-15.9	3.2	6.3	1.0
	Large	0								
	sp.	0								
Coxcatlan	Small	8	-9.1	2.5	-3.0	2.1	-18.5	1.7	7.6	1.3
	Large	1	-6.4		-6.0		-14.1		10.1	
	sp.	1	-11.0		-2.5		-18.5		4.5	
El Riego	Small	11	-8.2	2.8	-2.1	2.2	-16.4	2.8	9.0	1.9
	Large	5	-7.0	2.2	-1.9	1.9	-15.1	0.0	11.2	0.1
	sp.	1	-7.6		-3.3					
Ajuereado	Small	4	-9.2	0.8	-1.9	1.6				
	Large	13	-4.4	1.5	-2.3	1.4	-10.1	0.8	9.1	1.4
	sp.	0								

Table S4.

Descriptive statistics of the stable isotope values of rabbit (*Sylvilagus* spp.) specimens. Large morphotypes were identified as *Sylvilagus cunicularius* while small morphytypes likely represent either *Sylvilagus audubonii* or *Sylvilagus floridanus connectus.* Specimens identified as "sp." were identified only to genus level.

Comparison	Isotope	diff	lwr	upr	P adj	sig
Pleistocene-Early Holocene	$\delta^{13}C_{ap}$	3.2	1.4	4.9	0.0000	*
	$\delta^{13}C_{col}$	6.2	2.9	9.5	0.0000	*
	$\delta^{15}N_{col}$	-0.6	-2.9	1.7	0.8983	
	$\delta^{18}O_{ap}$	1.1	-0.7	3.0	0.4086	
Pleistocene-Mid Holocene	$\delta^{13}C_{ap}$	4.6	2.9	6.3	0.0000	*
	$\delta^{13}C_{col}$	6.7	3.4	10.0	0.0000	*
	$\delta^{15}N_{col}$	1.5	-0.8	3.8	0.3313	
	$\delta^{18}O_{ap}$	2.4	0.5	4.2	0.0052	*
Pleistocene-Late Holocene	$\delta^{13}C_{ap}$	3.4	1.8	4.9	0.0000	*
	$\delta^{13}C_{col}$	7.0	4.0	9.9	0.0000	*
	$\delta^{15}N_{col}$	2.4	0.3	4.4	0.0163	*
	$\delta^{18}O_{ap}$	2.3	0.7	4.0	0.0018	*
Mid Holocene-Early Holocene	$\delta^{13}C_{ap}$	-1.5	-2.9	0.0	0.0441	*
	$\delta^{13}C_{col}$	-0.5	-3.0	2.0	0.9606	
	$\delta^{15}N_{col}$	-2.1	-3.8	-0.3	0.0132	*
	$\delta^{18}O_{ap}$	-1.3	-2.8	0.3	0.1427	
Mid Holocene-Late Holocene	$\delta^{13}C_{ap}$	-1.2	-2.4	0.0	0.0439	*
	$\delta^{13}C_{col}$	0.3	-1.7	2.3	0.9806	
	$\delta^{15}N_{col}$	0.9	-0.5	2.3	0.3361	
	$\delta^{18}O_{ap}$	0.0	-1.3	1.2	0.9999	
Late Holocene-Early Holocene	$\delta^{13}C_{ap}$	-0.2	-1.5	1.0	0.9572	
	$\delta^{13}C_{col}$	-0.8	-2.8	1.3	0.7652	
	$\delta^{15}N_{col}$	-3.0	-4.4	-1.6	0.0000	*
	$\delta^{18}O_{ap}$	-1.2	-2.5	0.1	0.0738	

Table S5.

Results of Tukey Honest Significant Differences tests comparing combined deer and rabbit stable isotope values between geologic sub epochs. The differences between sub epoch means are indicated in the diff column. The lower and upper bounds of the 95% confidence intervals are indicated in columns lwr and upr columns, respectively. The Tukey-adjusted P-values is indicated in the P adj column. If the P value < 0.05, the row is highlighted with the * symbol in the sig column.

Source	Mean $\delta^{13}C$ ap (‰)	SD $\delta^{13}C$ ap (‰)	Mean $\delta^{18}O$ ap (‰)	SD $\delta^{18}O$ ap (‰)	Mean $\delta^{13}C$ col (‰)	SD $\delta^{13}C$ col (‰)	Mean $\delta^{15}N$ col (‰)	SD $\delta^{15}N$ col (‰)	n
Forest	-13	2	-4	2.5	-20	2.5	4	1.5	50
Grassland	-4	2	-2	2.5	-11.5	2.5	6	1.5	50
Desert	-9	2	-1	2.5	-16.1	2.5	9	1.5	50

Table S6.

Expected stable isotope values of generic environmental zones within the Tehuacan valley. Source values are based on values of similar environments across Mexico (*37*) and on archaeological rabbit specimens from similar environments (*43*). These data were the source values for the Bayesian stable isotope mixing model using the MixSIAR package.

Phase	Environment	Mean	SD	0.025	0.05	0.25	Median	0.75	0.95	0.975
Ajuereado	Desert	0.418	0.127	0.166	0.207	0.335	0.416	0.503	0.625	0.671
El Riego	Desert	0.879	0.077	0.707	0.735	0.831	0.889	0.938	0.984	0.992
Coxcatlan	Desert	0.536	0.16	0.229	0.277	0.427	0.532	0.646	0.801	0.854
Abejas	Desert	0.371	0.165	0.064	0.1	0.256	0.366	0.48	0.651	0.714
Purron	Desert	0.443	0.224	0.043	0.081	0.28	0.433	0.598	0.831	0.901
Ajalpan	Desert	0.506	0.236	0.063	0.112	0.326	0.508	0.681	0.89	0.936
Santa Maria	Desert	0.248	0.115	0.046	0.071	0.165	0.241	0.325	0.451	0.496
Palo Blanco	Desert	0.576	0.113	0.353	0.39	0.501	0.573	0.651	0.761	0.798
Venta Salada	Desert	0.333	0.109	0.122	0.152	0.262	0.331	0.403	0.518	0.561
Ajuereado	Forest	0.091	0.068	0.004	0.008	0.039	0.075	0.128	0.223	0.261
El Riego	Forest	0.066	0.053	0.002	0.004	0.024	0.053	0.095	0.171	0.198
Coxcatlan	Forest	0.346	0.154	0.051	0.095	0.238	0.346	0.451	0.606	0.652
Abejas	Forest	0.436	0.173	0.101	0.145	0.318	0.431	0.555	0.721	0.782
Purron	Forest	0.327	0.209	0.019	0.036	0.159	0.304	0.466	0.719	0.793
Ajalpan	Forest	0.346	0.227	0.018	0.03	0.157	0.317	0.508	0.773	0.855
Santa Maria	Forest	0.547	0.128	0.279	0.33	0.466	0.549	0.633	0.752	0.791
Palo Blanco	Forest	0.293	0.109	0.086	0.115	0.22	0.292	0.366	0.473	0.514
Venta Salada	Forest	0.491	0.112	0.268	0.304	0.417	0.493	0.568	0.672	0.703
Ajuereado	Grassland	0.491	0.127	0.228	0.281	0.409	0.494	0.577	0.696	0.733
El Riego	Grassland	0.055	0.045	0.002	0.003	0.021	0.044	0.08	0.14	0.166
Coxcatlan	Grassland	0.118	0.08	0.006	0.012	0.055	0.105	0.168	0.268	0.3
Abejas	Grassland	0.193	0.113	0.015	0.027	0.107	0.183	0.266	0.392	0.443
Purron	Grassland	0.23	0.168	0.01	0.021	0.096	0.198	0.327	0.558	0.635
Ajalpan	Grassland	0.148	0.123	0.005	0.01	0.053	0.119	0.207	0.389	0.461
Santa Maria	Grassland	0.206	0.092	0.029	0.051	0.142	0.203	0.268	0.363	0.393
Palo Blanco	Grassland	0.131	0.07	0.012	0.024	0.078	0.127	0.178	0.256	0.28
Venta Salada	Grassland	0.175	0.077	0.032	0.049	0.12	0.174	0.229	0.304	0.33

Table S7.

Posterior results of the MixSIAR model. The environmental data are grouped according to cultural phases. The numerical columns contain the mean, standard deviation (SD), and quantiles of the posterior distributions of each idealized environmental patch represented by the stable isotope values in rabbit bones.

Data S1. (separate file)

Supporting information for the Bayesian radiocarbon model of the Tehuacan Valley. (**A**) List of radiocarbon dates compiled for the model; (**B**) Posteriors from the Bayesian model; (**C**) OxCal 4.4 code; (**D**) Chronological information for each bone sample of study; (**D**) Relevant references.

Data S2. (separate file)

Stable isotope data from Tehuacan Valley animal bone specimens presented with specimen information, diagenesis data, and contextual information.

REFERENCES

1. Y. Matsuoka, Y. Vigouroux, M. M. Goodman, G. J. Sanchez, E. Buckler, J. Doebley, A single domestication for maize shown by multilocus microsatellite genotyping. *Proc. Natl. Acad. Sci. U.S.A.* **99**, 6080–6084 (2002).

2. D. R. Piperno, A. J. Ranere, I. Holst, J. Iriarte, R. Dickau, Starch grain and phytolith evidence for early ninth millennium B.P. maize from the Central Balsas River Valley, Mexico. *Proc. Natl. Acad. Sci. U.S.A.* **106**, 5019–5024 (2009).

3. M. E. Pohl, D. R. Piperno, K. O. Pope, J. G. Jones, Microfossil evidence for pre-Columbian maize dispersals in the neotropics from San Andres, Tabasco, Mexico. *Proc. Natl. Acad. Sci. U.S.A.* **104**, 6870–6875 (2007).

4. A. Grobman, D. Bonavia, T. D. Dillehay, D. R. Piperno, J. Iriarte, I. Holst, Preceramic maize from Paredones and Huaca Prieta, Peru. *Proc. Natl. Acad. Sci. U.S.A.* **109**, 1755–1759 (2012).

5. J. M. Vint, "Las Capas, AZ AA:12:111 (ASM), Introduced: Background, chronology, and research orientation," in *Implements of Change: Tools, Subsistence, and the Built Environment of Las Capas, an Early Agricultural Irrigation Community in Southern Arizona* (Anthropological Papers No. 51. Archaeology Southwest, Tucson), pp. 1–31.

6. N. Yang, Y. Wang, X. Liu, M. Jin, M. Vallebueno-Estrada, E. Calfee, L. Chen, B. P. Dilkes, S. Gui, X. Fan, T. K. Harper, D. J. Kennett, W. Li, Y. Lu, J. Ding, Z. Chen, J. Luo, S. Mambakkam, M. Menon, S. Snodgrass, C. Veller, S. Wu, S. Wu, L. Zhuo, Y. Xiao, X. Yang, M. C. Stitzer, D. Runcie, J. Yan, J. Ross-Ibarra, Two teosintes made modern maize. *Science* **382**, eadg8940 (2023).

7. M. B. Hufford, P. Lubinksy, T. Pyhäjärvi, M. T. Devengenzo, N. C. Ellstrand, J. Ross-Ibarra, The genomic signature of crop-wild introgression in maize. *PLOS Genet.* **9**, e1003477 (2013).

8. E. Calfee, D. Gates, A. Lorant, M. T. Perkins, G. Coop, J. Ross-Ibarra, Selective sorting of ancestral introgression in maize and teosinte along an elevational cline. *PLOS Genet.* **17**, e1009810 (2021).

9. M. Blake, *Maize for the Gods: Unearthing the 9,000-Year History of Corn* (University of California Press, Oakland, 2015).

10. D. J. Kennett, K. M. Prufer, B. J. Culleton, R. J. George, M. Robinson, W. R. Trask, G. M. Buckley, E. Moes, E. J. Kate, T. K. Harper, Early isotopic evidence for maize as a staple grain in the Americas. *Sci. Adv.* **6**, eaba3245 (2020).

11. R. M. Rosenswig, Opinions on the Lowland Maya Late Archaic period with some evidence from northern Belize. *Anc. Mesoam.* **32**, 461–474 (2021).

12. P. J. Richerson, R. Boyd, R. L. Bettinger, Was agriculture impossible during the Pleistocene but mandatory during the Holocene? A climate change hypothesis. *Am. Antiq.* **66**, 387–411 (2001).

13. V. G. Childe, *Man Makes Himself* (Mentor Books, 1951).

14. M. N. Cohen, *Food Crisis in Prehistory: Overpopulation and the Origins of Agriculture* (Yale Univ. Press, New Haven, 1977), vol. 3, p. 332.

15. H. Weiss, R. S. Bradley, What drives societal collapse? *Science* **291**, 609 (2001).

16. B. D. Smith, A comparison of niche construction theory and diet breadth models as explanatory frameworks for the initial domestication of plants and animals. *J. Archeol. Res.* **23**, 215–262 (2015).

17. G. Willcox, The distribution, natural habitats and availability of wild cereals in relation to their domestication in the Near East: Multiple events, multiple centres. *Veg. Hist. Archaeobotany* **14**, 534–541 (2005).

18. M. A. Zeder, The broad spectrum revolution at 40: Resource diversity, intensification, and an alternative to optimal foraging explanations. *J. Anthropol. Archaeol.* **31**, 241–264 (2012).

19. B. F. Benz, L. Cheng, S. W. Leavitt, C. Eastoe, "El Riego and early maize agricultural evolution," in *Histories of Maize*, J. Staller, R. Tykot, B. Benz, Eds. (Elsevier, New York, 2006), pp. 73–82.

20. B. D. Smith, Reassessing Coxcatlan Cave and the early history of domesticated plants in Mesoamerica. *Proc. Natl. Acad. Sci. U.S.A.* **102**, 9438–9445 (2005).

21. C. E. Smith, "Plant remains," in *The Prehistory of the Tehuacan* Valley, D. S. Byers, Ed. (University of Texas Press, Austin, 1967), vol. 1, pp. 220–255.

22. M. Vallebueno-Estrada, I. Rodríguez-Arévalo, A. Rougon-Cardoso, J. Martínez González, A. García Cook, R. Montiel, J.-P. Vielle-Calzada, The earliest maize from San Marcos Tehuacán is a partial domesticate with genomic evidence of inbreeding. *Proc. Natl. Acad. Sci. U.S.A.* **113**, 14151–14156 (2016).

23. A. Long, B. F. Benz, D. J. Donahue, A. J. T. Jull, L. J. Toolin, First direct AMS dates on early maize from Tehuacán, Mexico. *Radiocarbon* **31**, 1035–1040 (1989).

24. E. Torres-Rodríguez, M. Vallebueno-Estrada, J. M. González, A. G. Cook, R. Montiel, J.-P. Vielle-Calzada, AMS dates of new maize specimens found in rock shelters of the Tehuacan Valley. *Radiocarbon* **60**, 975–987 (2018).

25. J. A. Neely, M. J. Aiuvalasit, B. M. Winsborough, Relict canals of the Tehuacán Valley, Mexico: A middle-to late-holocene dryland socio-hydrological system. *Holocene* **32**, 1422–1436 (2022).

26. R. D. Drennan, M. J. Haller, "The local village community and the larger political economy: Formative and Classic interaction patterns in the Tehuacán Valley compared to the Valley of Oaxaca and the Basin of Mexico," in *The Political Economy of Ancient Mesoamerica: Transformations during the Formative and Classic Periods*, V. L. Scarborough, J. E. Clark, Eds. (University of New Mexico Press, Albuquerque, 2007), pp. 65–81.

27. R. S. MacNeish, Ancient mesoamerican civilization. *Science* **143**, 531–537 (1964).

28. C. S. Spencer, Human agency, biased transmission, and the cultural evolution of chiefly authority. *J. Anthropol. Archaeol.* **12**, 41–74 (1993).

29. M. J. DeNiro, S. Epstein, Influence of diet on the distribution of carbon isotopes in animals. *Geochim. Cosmochim. Acta* **42**, 495–506 (1978).

30. M. J. DeNiro, S. Epstein, Influence of diet on the distribution of nitrogen isotopes in animals. *Geochim. Cosmochim. Acta* **45**, 341–351 (1981).

31. A. Longinelli, Oxygen isotopes in mammal bone phosphate: A new tool for paleohydrological and paleoclimatological Research? *Geochim. Cosmochim. Acta* **48**, 385–390 (1984).

32. S. H. Ambrose, Effects of diet, climate and physiology on nitrogen isotope abundances in terrestrial foodwebs. *J. Archaeol. Sci.* **18**, 293–317 (1991).

33. G. Hartman, Are elevated δ15N values in herbivores in hot and arid environments caused by diet or animal physiology? *Funct. Ecol.* **25**, 122–131 (2011).

34. A. D. Huertas, P. Iacumin, B. Stenni, B. S. Chillon, A. Longinelli, Oxygen isotope variations of phosphate in mammalian bone and tooth enamel. *Geochim. Cosmochim. Acta* **59**, 4299–4305 (1995).

35. K. A. Hallin, M. J. Schoeninger, H. P. Schwarcz, Paleoclimate during Neandertal and anatomically modern human occupation at Amud and Qafzeh, Israel: The stable isotope data. *J. Hum. Evol.* **62**, 59–73 (2012).

36. B. P. Murphy, D. M. J. S. Bowman, The carbon and nitrogen isotope composition of Australian grasses in relation to climate. *Funct. Ecol.* **23**, 1040–1049 (2009).

37. A. D. Somerville, A. W. Froehle, M. J. Schoeninger, Environmental influences on rabbit and hare bone isotope abundances: Implications for paleoenvironmental research. *Palaeogeogr. Palaeoclimatol. Palaeoecol.* **497**, 91–104 (2018).

38. F. Johnson, R. S. MacNeish, "Chronometric dating," in *The Prehistory of the Tehuacán Valley: Chronology and Irrigation*, R. S. MacNeish, Ed. (University of Texas Press, Austin, 1972), vol. 4, pp. 3–55.

39. R. S. MacNeish, "An interdisciplinary approach to an archaeological problem" in *Prehistory of the Tehuacan Valley. Environment and Subsistence* (University of Texas Press, Austin, 1967), vol. 1, pp. 14–24.

40. A. D. Somerville, I. Casar, J. Arroyo-Cabrales, New AMS radiocarbon ages from the Preceramic levels of Coxcatlan Cave, Puebla, Mexico: A Pleistocene occupation of the Tehuacan Valley? *Lat. Am. Antiq.* **32**, 612–626 (2021).

41. A. T. Smith, C. H. Johnston, P. C. Alves, K. Hackländer, *Lagomorphsyikas, Rabbits, and Hares of the World* (JHU Press, 2018).

42. M. J. Walker, M. Berkelhammer, S. Björck, L. C. Cwynar, D. A. Fisher, A. J. Long, J. J. Lowe, R. M. Newnham, S. O. Rasmussen, H. Weiss, Formal subdivision of the Holocene Series/Epoch: A discussion paper by a working group of INTIMATE (Integration of ice-core, marine and terrestrial records) and the Subcommission on Quaternary Stratigraphy (International Commission on Stratigraphy). *J. Quat. Sci.* **27**, 649–659 (2012).

43. A. D. Somerville, B. A. Nelson, J. L. Punzo, M. J. Schoeninger, Rabbit bone stable isotope values distinguish desert ecoregions of North America: Data from the archaeological sites of Pueblo Grande, La Ferreria, and La Quemada. *J. Archaeol. Sci.* **113**, 105063 (2020).

44. P. M. Grootes, M. Stuiver, J. W. C. White, S. Johnsen, J. Jouzel, Comparison of oxygen isotope records from the GISP2 and GRIP Greenland ice cores. *Nature* **366**, 552–554 (1993).

45. B. M. Vinther, S. L. Buchardt, H. B. Clausen, D. Dahl-Jensen, S. J. Johnsen, D. A. Fisher, R. M. Koerner, D. Raynaud, V. Lipenkov, K. K. Andersen, Holocene thinning of the Greenland ice sheet. *Nature* **461**, 385–388 (2009).

46. M. Caballero, S. Lozano-García, B. Ortega-Guerrero, A. Correa-Metrio, Quantitative estimates of orbital and millennial scale climatic variability in central Mexico during the last □40,000 years. *Quat. Sci. Rev.* **205**, 62–75 (2019).

47. M. Caballero, M. de S. Lozano-García, B. Ortega-Guerrero, “Paleoenvironmental change in Central Mexico during the last 20,000 years,” in *Lake Alchichica Limnology: The Uniqueness of a Tropical Maar Lake* (Springer, 2022), pp. 33–50.

48. R. B. Alley, P. A. Mayewski, T. Sowers, M. Stuiver, K. C. Taylor, P. U. Clark, Holocene climatic instability: A prominent, widespread event 8200 yr ago. *Geology* **25**, 483–486 (1997).

49. P. A. Mayewski, E. E. Rohling, J. C. Stager, W. Karlen, K. A. Maasch, L. D. Meeker, E. Meyerson, F. Gasse, S. van Kreveld, K. Holmgren, J. Lee-Thorp, G. Rosqvist, F. Rack, M. Staubwasser, R. R. Schneider, E. J. Steig, Holocene climate variability. *Quatern. Res.* **62**, 243–255 (2004).

50. J. P. Bernal, M. Lachniet, M. McCulloch, G. Mortimer, P. Morales, E. Cienfuegos, A speleothem record of Holocene climate variability from southwestern Mexico. *Quatern. Res.* **75**, 104–113 (2011).

51. D. S. Byers, *The Prehistory of the Tehuacan Valley: Environment and Subsistence* (University of Texas Press, 1967), vol. 1.

52. G. H. Haug, K. A. Hughen, D. M. Sigman, L. C. Peterson, U. Röhl, Southward migration of the intertropical convergence zone through the Holocene. *Science* **293**, 1304–1308 (2001).

53. H. Renssen, H. Seppä, X. Crosta, H. Goosse, D. M. Roche, Global characterization of the holocene thermal maximum. *Quat. Sci. Rev.* **48**, 7–19 (2012).

54. D. A. Hodell, J. H. Curtis, M. Brenner, Possible role of climate in the collapse of classic maya civilization. *Nature* **375**, 391–394 (1995).

55. J. L. Conroy, J. T. Overpeck, J. E. Cole, T. M. Shanahan, M. Steinitz-Kannan, Holocene changes in eastern tropical Pacific climate inferred from a Galápagos lake sediment record. *Quat. Sci. Rev.* **27**, 1166–1180 (2008).

56. V. O. Magaña, J. L. Vázquez, J. L. Pérez, J. B. Pérez, Impact of El Niño on precipitation in Mexico. *Geofís. Int.* **42**, 313–330 (2003).

57. D. W. Stahle, E. R. Cook, D. J. Burnette, J. Villanueva, J. Cerano, J. N. Burns, D. Griffin, B. I. Cook, R. Acuña, M. C. A. Torbenson, P. Szejner, I. M. Howard, The Mexican drought

Atlas: Tree-ring reconstructions of the soil moisture balance during the late pre-Hispanic, colonial, and modern eras. *Quat. Sci. Rev.* **149**, 34–60 (2016).

58. H. W. Arz, F. Lamy, J. Pätzold, A pronounced dry event recorded around 4.2 ka in brine sediments from the northern Red Sea. *Quatern. Res.* **66**, 432–441 (2006).

59. R. K. Booth, S. T. Jackson, S. L. Forman, J. E. Kutzbach, E. A. B. Iii, J. Kreigs, D. K. Wright, A severe centennial-scale drought in midcontinental North America 4200 years ago and apparent global linkages. *Holocene* **15**, 321–328 (2005).

60. H. Weiss, M.-A. Courty, W. Wetterstrom, F. Guichard, L. Senior, R. Meadow, A. Curnow, The genesis and collapse of third millennium north Mesopotamian civilization. *Science* **261**, 995–1004 (1993).

61. M. S. Lachniet, Y. Asmerom, J. P. Bernal, V. J. Polyak, L. Vazquez-Selem, Orbital pacing and ocean circulation-induced collapses of the Mesoamerican monsoon over the past 22,000 y. *Proc. Natl. Acad. Sci. U.S.A.* **110**, 9255–9260 (2013).

62. S. E. Metcalfe, S. J. Davies, J. D. Braisby, M. J. Leng, A. J. Newton, N. L. Terrett, S. L. O'Hara, Long and short-term change in the Pátzcuaro Basin, central Mexico. *Palaeogeogr. Palaeoclimatol. Palaeoecol.* **247**, 272–295 (2007).

63. T. Bhattacharya, R. Byrne, H. Böhnel, K. Wogau, U. Kienel, B. L. Ingram, S. Zimmerman, Cultural implications of late Holocene climate change in the Cuenca Oriental, Mexico. *Proc. Natl. Acad. Sci. U.S.A.* **112**, 1693–1698 (2015).

64. D. J. Kennett, S. F. M. Breitenbach, V. V. Aquino, Y. Asmerom, J. Awe, J. U. L. Baldini, P. Bartlein, B. J. Culleton, C. Ebert, C. Jazwa, M. J. Macri, N. Marwan, V. Polyak, K. M. Prufer, H. E. Ridley, H. Sodemann, B. Winterhalder, G. H. Haug, Development and disintegration of Maya political systems in response to climate change. *Science* **338**, 788–791 (2012).

65. J. Park, R. Byrne, H. Böhnel, Late Holocene climate change in Central Mexico and the decline of Teotihuacan. *Ann. Am. Assoc. Geogr.* **109**, 104–120 (2019).

66. K. V. Flannery, “Vertebrate fauna and hunting patterns,” *in The Prehistory of the Tehuacan Valley*, D. S. Byers, Ed. (University of Texas Press, Austin, 1967), vol. 1, pp. 132–177.

67. C. C. Fennell, Assessing criticisms of faunal analyses and environmental reconstructions in the Tehuacan Valley Project. *Hum. Ecol.* **29**, 349–359 (2001).

68. D. R. Piperno, K. V. Flannery, The earliest archaeological maize (Zea mays L.) from highland Mexico: New accelerator mass spectrometry dates and their implications. *Proc. Natl. Acad. Sci. U.S.A.* **98**, 2101–2103 (2001).

69. K. V. Flannery, *Guila Naquitz: Archaic Foraging and Early Agriculture in Oaxaca, Mexico* (Academic Press, Inc., Orlando, 1986).

70. M. C. Stitzer, J. Ross-Ibarra, Maize domestication and gene interaction. *New Phytol.* **220**, 395–408 (2018).

71. I. Lopez-Valdivia, A. C. Perkins, H. M. Schneider, M. Vallebueno-Estrada, J. D. Burridge, E. González-Orozco, A. Montufar, R. Montiel, J. P. Lynch, J.-P. Vielle-Calzada, Gradual domestication of root traits in the earliest maize from Tehuacán. *Proc. Natl. Acad. Sci. U.S.A.* **119**, e2110245119 (2022).

72. I. Lopez-Valdivia, M. Vallebueno-Estrada, H. Rangarajan, K. Swarts, B. F. Benz, M. Blake, J. S. Sidhu, S. Perez-Limon, R. J. Sawers, H. Schneider, In silico analysis of the evolution of root phenotypes during maize domestication in Neolithic soils of Tehuacán. *New Phytol.* **248**, 339–353 (2025).

73. B. F. Benz, Archaeological evidence of teosinte domestication from Guilá Naquitz, Oaxaca. *Proc. Natl. Acad. Sci. U.S.A.* **98**, 2104–2106 (2001).

74. P. C. Mangelsdorf, “Prehistoric wild and cultivated maize,” in *The Prehistory of the Tehuacan Valley*, D. S. Byers, Ed. (University of Texas Press, Austin, 1967), vol. 1, pp. 178–200.

75. B. F. Benz, H. H. Iltis, Studies in archaeological maize I: The “wild” maize from San Marcos Cave reexamined. *Am. Antiq.* **55**, 500–511 (1990).

76. J. Ramos-Madrigal, B. D. Smith, J. V. Moreno-Mayar, S. Gopalakrishnan, J. Ross-Ibarra, M. T. P. Gilbert, N. Wales, Genome sequence of a 5,310-year-old maize cob provides insights into the early stages of maize domestication. *Curr. Biol.* **26**, 3195–3201 (2016).

77. M. Vallebueno-Estrada, G. G. Hernández-Robles, E. González-Orozco, I. López-Valdivia, T. R. Tham, V. V. Sánchez, K. Swarts, T. D. Dillehay, J.-P. Vielle-Calzada, R. Montiel, Domestication and lowland adaptation of coastal preceramic maize from Paredones, Peru. *eLife* **12**, e83149 (2023).

78. B. D. Smith, Low-level food production. *J. Archeol. Res.* **9**, 1–43 (2001).

79. R. S. MacNeish, F. A. Peterson, K. V. Flannery, *The Prehistory of the Tehuacan Valley: Vol 5: Excavations and Reconnaissance* (University of Texas Press, Austin, 1972).

80. R. M. Rosenswig, A mosaic of adaptation: The archaeological record for Mesoamerica's Archaic period. *J. Archeol. Res.* **23**, 115–162 (2015).

81. J. E. Clark, M. Blake, "The power of prestige: Competitive generosity and the emergence of rank societies in lowland Mesoamerica," in *Factional Competition and Political Development in the New World*, E. M. Brumfiel, J. W. Fox, Eds. (Cambridge Univ. Press, Cambridge, 1994), *New Directions in Archaeology*, pp. 17–30; https://www.cambridge.org/core/product/2FAABEAD2F4516969D4DBE603C15F5A5.

82. L. Kaplan, T. F. Lynch, Phaseolus (Fabaceae) in archaeology: AMS radiocarbon dates and their significance for Pre-Colombian agriculture. *Econ. Bot.* **53**, 261–272 (1999).

83. M. M. Beasley, E. J. Bartelink, L. Taylor, R. M. Miller, Comparison of transmission FTIR, ATR, and DRIFT spectra: Implications for assessment of bone bioapatite diagenesis. *J. Archaeol. Sci.* **46**, 16–22 (2014).

84. M. J. DeNiro, Postmortem preservation and alteration of in vivo bone collagen isotope ratios in relation to palaeodietary reconstruction. *Nature* **317**, 806–809 (1985).

85. A. von den Driesch, *A Guide to the Measurement of Animal Bones from Archaeological Sites: As Developed by the Institut Für Palaeoanatomie, Domestikationsforschung Und Geschichte*

Der Tiermedizin of the University of Munich (Peabody Museum Press, Harvard University, Cambridge, 1976), vol. 1.

86. P. L. Koch, N. Tuross, M. L. Fogel, The effects of sample treatment and diagenesis on the isotopic integrity of carbonate in biogenic hydroxylapatite. *J. Archaeol. Sci.* **24**, 417–429 (1997).

87. T. B. Coplen, W. A. Brand, M. Gehre, M. Gröning, H. A. J. Meijer, B. Toman, R. M. Verkouteren, New guidelines for δ 13C measurements. *Anal. Chem.* **78**, 2439–2441 (2006).

88. J. Sealy, M. Johnson, M. Richards, O. Nehlich, Comparison of two methods of extracting bone collagen for stable carbon and nitrogen isotope analysis: Comparing whole bone demineralization with gelatinization and ultrafiltration. *J. Archaeol. Sci.* **47**, 64–69 (2014).

89. S. Weiner, O. Bar-Yosef, States of preservation of bones from prehistoric sites in the Near East: A survey. *J. Archaeol. Sci.* **17**, 187–196 (1990).

90. L. E. Wright, H. P. Schwarcz, Infrared and isotopic evidence for diagenesis at Dos Pilas, Guatemala: Palaeodietary implications. *J. Archaeol. Sci.* **23**, 933–944 (1996).

91. R Core Team, R: A language and environment for statistical computing (2021); https://www.R-project. org.

92. B. C. Stock, A. L. Jackson, E. J. Ward, A. C. Parnell, D. L. Phillips, B. X. Semmens, Analyzing mixing systems using a new generation of Bayesian tracer mixing models. *PeerJ* **6**, e5096 (2018).

93. A. Ugan, J. Coltrain, Variation in collagen stable nitrogen values in black-tailed jackrabbits (*Lepus californicus*) in relation to small-scale differences in climate, soil, and topography. *J. Archaeol. Sci.* **38**, 1417–1429 (2011).

94. C. M. Munoz, R. Mauldin, D. Paul, L. Kemp, Monitoring paleovegetation shifts through stable carbon isotope variablity in archaeologically recovered leporids. *Texas J. Sci.* **63**, 113 (2011).

95. A. M. VanDerwarker, H. B. Thakar, K. Hirth, A. I. Domic, T. K. Harper, R. J. George, E. S. Johnson, V. Newhall, T. E. Scheffler, W. C. McCool, Early evidence of avocado domestication from El Gigante Rockshelter, Honduras. *Proc. Natl. Acad. Sci. U.S.A.* **122**, e2417072122 (2025).

96. C. Bronk Ramsey, Bayesian analysis of radiocarbon dates. *Radiocarbon* **51**, 337–360 (2009).

97. P. J. Reimer, W. E. N. Austin, E. Bard, A. Bayliss, P. G. Blackwell, C. B. Ramsey, M. Butzin, H. Cheng, R. L. Edwards, M. Friedrich, P. M. Grootes, T. P. Guilderson, I. Hajdas, T. J. Heaton, A. G. Hogg, K. A. Hughen, B. Kromer, S. W. Manning, R. Muscheler, J. G. Palmer, C. Pearson, J. van der Plicht, R. W. Reimer, D. A. Richards, E. M. Scott, J. R. Southon, C. S. M. Turney, L. Wacker, F. Adolphi, U. Büntgen, M. Capano, S. M. Fahrni, A. Fogtmann-Schulz, R. Friedrich, P. Köhler, S. Kudsk, F. Miyake, J. Olsen, F. Reinig, M. Sakamoto, A. Sookdeo, S. Talamo, The IntCal20 northern hemisphere radiocarbon age calibration curve (0–55 cal kBP). *Radiocarbon* **62**, 725–757 (2020).

98. P. L. Koch, Isotopic reconstruction of past continental environments. *Annu. Rev. Earth Planet. Sci.* **26**, 573–613 (1998).

99. M. J. Kirkby, A. V. Whyte, K. V. Flannery, "The physical environment of the Guilá Naquitz Cave group," in *Guilá Naquitz: Archaic Foraging and Early Agriculture in Oaxaca, Mexico* (Academic Press, Inc., Orlando, 2021), pp. 43–61.

100. J. A. Chapman, G. Ceballos, "The cottontails," *in Rabbits, Hares and Pikas: Status Survey and Conservation Action Plan*, J. A. Chapman, J. E. C. Flux, Eds. (International Union for the Conservation of Nature and Natural Resources, Gland, Switzerland, 1990), pp. 95–110.

101. Y. Vasquez, L. Tarango, E. López-Pérez, J. Herrera, G. Mendoza, S. Mandujano, Variation in the diet composition of the white tailed deer (*Odocoileus virginianus*) in the Tehuacán-Cuicatlán biosphere reserve. *Rev. Chapingo Ser. Cienc. For. Ambient.* **22**, 87–98 (2016).

102. L. K. Halls, *White-Tailed Deer: Ecology and Management* (Stackpole, Harrisburg, PA, 1984).

103. W. P. Smith, Odocoileus virginianus. *Mammalian Species* **388**, 1–13 (1991).

104. T. B. Bobby, B. D. Leopold, L. W. B. Jr, K. D. Godwin, Movements and home range dynamics of cottontail rabbits in Mississippi. *J. Wildl. Manage.* **65**, 1004–1013 (2001).

105. R. K. Swihart, Home range-body mass allometry in rabbits and hares (*Leporidae*). *Acta Theriol.* **31**, 139–148 (1986).

106. T. T. Trent, O. J. Rongstad, Home range and survival of cottontail rabbits in southwestern Wisconsin. *J. Wildl. Manage.* **38**, 459–472 (1974).

107. G. B. Rose, Mortality rates of tagged adult cottontail rabbits. *J. Wildl. Manage.* **41**, 511–514 (1977).

108. M. J. Schoeninger, M. J. DeNiro, Nitrogen and carbon isotopic composition of bone collagen from marine and terrestrial animals. *Geochim. Cosmochim. Acta* **48**, 625–639 (1984).

109. M. M. Bender, Mass spectrometric studies of carbon 13 variations in corn and other grasses. *Radiocarbon* **10**, 468–472 (1968).

110. B. N. Smith, S. Epstein, Two categories of 13C/12C ratios for higher plants. *Plant Physiol.* **47**, 380–384 (1971).

111. M. J. Kohn, Carbon isotope compositions of terrestrial C3 plants as indicators of (paleo) ecology and (paleo)climate. *Proc. Natl. Acad. Sci. U.S.A.* **107**, 19691–19695 (2010).

112. M. H. O'Leary, Carbon isotopes in photosynthesis. *Bioscience* **38**, 328–336 (1988).

113. L. G. Stowe, J. A. Teeri, The geographic distribution of C4 species of the dicotyledonae in relation to climate. *Am. Nat.* **112**, 609–623 (1978).

114. L. L. Tieszen, Natural variations in the carbon isotope values of plants: Implications for archaeology, ecology, and paleoecology. *J. Archaeol. Sci.* **18**, 227–248 (1991).

115. G. D. Farquhar, J. R. Ehleringer, K. T. Hubick, Carbon isotope discrimination and photosynthesis. *Annu. Rev. Plant Biol.* **40**, 503–537 (1989).

116. M. Bonafini, M. Pellegrini, P. Ditchfield, A. M. Pollard, Investigation of the 'canopy effect' in the isotope ecology of temperate woodlands. *J. Archaeol. Sci.* **40**, 3926–3935 (2013).

117. N. J. van der Merwe, E. Medina, The canopy effect, carbon isotope ratios and foodwebs in amazonia. *J. Archaeol. Sci.* **18**, 249–259 (1991).

118. J. C. Vogel, Recycling of carbon in a forest environment. *Oecol. Plantar.* **13**, 89–94 (1978).

119. S. H. Ambrose, L. Norr, "Experimental evidence for the relationship of the carbon isotope ratios of whole diet and dietary protein to those of bone collagen and carbonate," in *Prehistoric Human Bone: Archaeology at the Molecular Level* (Springer, New York, 1993), pp. 1–37.

120. K. F. Emery, L. E. Wright, H. Schwarcz, Isotopic analysis of ancient deer bone: Biotic stability in collapse period maya land-use. *J. Archaeol. Sci.* **27**, 537–550 (2000).

121. A. W. Froehle, C. M. Kellner, M. J. Schoeninger, FOCUS: Effect of diet and protein source on carbon stable isotope ratios in collagen: Follow up to Warinner and Tuross (2009). *J. Archaeol. Sci.* **37**, 2662–2670 (2010).

122. D. J. Bryant, P. L. Koch, P. N. Froelich, W. J. Showers, B. J. Genna, Oxygen isotope partitioning between phosphate and carbonate in mammalian apatite. *Geochim. Cosmochim. Acta* **60**, 5145–5148 (1996).

123. P. Iacumin, H. Bocherens, A. Mariotti, A. Longinelli, Oxygen isotope analyses of co-existing carbonate and phosphate in biogenic apatite: A way to monitor diagenetic alteration of bone phosphate? *Earth Planet. Sci. Lett.* **142**, 1–6 (1996).

124. B. Luz, Y. Kolodny, M. Horowitz, Fractionation of oxygen isotopes between mammalian bone-phosphate and environmental drinking water. *Geochim. Cosmochim.* **48**, 1689–1693 (1984).

125. W. Dansgaard, Stable isotopes in precipitation. *Tellus* **16**, 436–468 (1964).

126. H. C. Fricke, J. R. O'Neil, The correlation between 18O/16O ratios of meteoric water and surface temperature: Its use in investigating terrestrial climate change over geologic time. *Earth Planet. Sci. Lett.* **170**, 181–196 (1999).

127. M. A. Poage, C. P. Chamberlain, Empirical relationships between elevation and the stable isotope composition of precipitation and surface waters: Considerations for studies of paleoelevation change. *Am. J. Sci.* **301**, 1–15 (2001).

128. L. K. Ayliffe, A. R. Chivas, Oxygen isotope composition of the bone phosphate of Australian kangaroos: Potential as a palaeoenvironmental recorder. *Geochim. Cosmochim.* **54**, 2603–2609 (1990).

129. N. E. Levin, T. E. Cerling, B. H. Passey, J. M. Harris, J. R. Ehleringer, A stable isotope aridity index for terrestrial environments. *Proc. Natl. Acad. Sci. U.S.A.* **103**, 11201–11205 (2006).

130. R. L. Burk, M. Stuiver, Oxygen isotope ratios in trees reflect mean annual temperature and humidity. *Science* **211**, 1417–1419 (1981).

131. D. Robinson, δ15N as an integrator of the nitrogen cycle. *Trends Ecol. Evol.* **16**, 153–162 (2001).

132. Q. Wang, J. Liu, H. Zhu, Genetic and molecular mechanisms underlying symbiotic specificity in legume-rhizobium interactions. *Front. Plant Sci.* **9**, 313 (2018).

133. R. Amundson, A. T. Austin, E. A. G. Schuur, K. Yoo, V. Matzek, C. Kendall, A. Uebersax, D. Brenner, W. T. Baisden, Global patterns of the isotopic composition of soil and plant nitrogen. *Global Biogeochem. Cycles* **17**, 1031 (2003).

134. J. M. Craine, A. J. Elmore, L. Wang, L. Augusto, W. T. Baisden, E. N. J. Brookshire, M. D. Cramer, N. J. Hasselquist, E. A. Hobbie, A. Kahmen, K. Koba, J. M. Kranabetter, M. C. Mack, E. Marin-Spiotta, J. R. Mayor, K. K. McLauchlan, A. Michelsen, G. B. Nardoto, R. S. Oliveira, S. S. Perakis, P. L. Peri, C. A. Quesada, A. Richter, L. A. Schipper, B. A.

Stevenson, B. L. Turner, R. A. G. Viani, W. Wanek, B. Zeller, Convergence of soil nitrogen isotopes across global climate gradients. *Sci. Rep.* **5**, 8280 (2015).

135. J. M. Craine, A. J. Elmore, M. P. M. Aidar, M. Bustamante, T. E. Dawson, E. A. Hobbie, A. Kahmen, M. C. Mack, K. K. McLauchlan, A. Michelsen, G. B. Nardoto, L. H. Pardo, J. Peñuelas, P. B. Reich, E. A. G. Schuur, W. D. Stock, P. H. Templer, R. A. Virginia, J. M. Welker, I. J. Wright, Global patterns of foliar nitrogen isotopes and their relationships with climate, mycorrhizal fungi, foliar nutrient concentrations, and nitrogen availability. *New Phytol.* **183**, 980–992 (2009).

136. A. T. Austin, P. M. Vitousek, Nutrient dynamics on a precipitation gradient in Hawai'i. *Oecologia* **113**, 519–529 (1998).

137. J. M. Craine, E. N. J. Brookshire, M. D. Cramer, N. J. Hasselquist, K. Koba, E. Marin-Spiotta, L. Wang, Ecological interpretations of nitrogen isotope ratios of terrestrial plants and soils. *Plant and Soil* **396**, 1–26 (2015).

138. D. R. Gröcke, H. Bocherens, A. Mariotti, Annual rainfall and nitrogen-isotope correlation in macropod collagen: Application as a palaeoprecipitation indicator. *Earth Planet. Sci. Lett.* **153**, 279–285 (1997).

139. B. P. Murphy, D. M. J. S. Bowman, Kangaroo metabolism does not cause the relationship between bone collagen δ15N and water availability. *Funct. Ecol.* **20**, 1062–1069 (2006).

140. G. Hartman, A. Danin, Isotopic values of plants in relation to water availability in the Eastern Mediterranean region. *Oecologia* **162**, 837–852 (2010).
